# Gluconate suppresses seizure activity in developing brains by inhibiting CLC-3 chloride channels

**DOI:** 10.1186/s13041-019-0465-0

**Published:** 2019-05-15

**Authors:** Zheng Wu, Qingwei Huo, Liang Ren, Fengping Dong, Mengyang Feng, Yue Wang, Yuting Bai, Bernhard Lüscher, Sheng-Tian Li, Guan-Lei Wang, Cheng Long, Yun Wang, Gangyi Wu, Gong Chen

**Affiliations:** 1 Department of Biology, Huck Institutes of Life Sciences, The Pennsylvania State University, University Park, PA 16802 USA; 2 School of Life Sciences, South China Normal University, Guangzhou, 510631 China; 3 Institutes of Brain Science, Fudan University, Shanghai, 200032 China; 4 Bio-X Institutes, Shanghai Jiao Tong University, 800 Dongchuan Road, Shanghai, 200240 China; 5 Department of Pharmacology, Zhongshan School of Medicine, Sun Yat-sen University, Guangzhou, 510080 China; 6 South China Research Center for Acupuncture-Moxibustion, Medical College of Acupuncture-Moxibustion and Rehabilitation, Guangzhou Univ Chinese Med, Guangzhou, 510006 China

**Keywords:** Neonatal seizure, Epilepsy, Gluconate, Anticonvulsant, CLC-3, Chloride channels, GABA

## Abstract

Neonatal seizures are different from adult seizures, and many antiepileptic drugs that are effective in adults often fail to treat neonates. Here, we report that gluconate inhibits neonatal seizure by inhibiting CLC-3 chloride channels. We detect a voltage-dependent outward rectifying Cl^−^ current mediated by CLC-3 Cl^−^ channels in early developing brains but not adult mouse brains. Blocking CLC-3 Cl^−^ channels by gluconate inhibits seizure activity both in neonatal brain slices and in neonatal animals with in vivo EEG recordings. Consistently, neonatal neurons of CLC-3 knockout mice lack the outward rectifying Cl^−^ current and show reduced epileptiform activity upon stimulation. Mechanistically, we demonstrate that activation of CLC-3 Cl^−^ channels alters intracellular Cl^−^ homeostasis and enhances GABA excitatory activity. Our studies suggest that gluconate can suppress neonatal seizure activities through inhibiting CLC-3 Cl^−^ channels in developing brains.

**Electronic supplementary material:**

The online version of this article (10.1186/s13041-019-0465-0) contains supplementary material, which is available to authorized users.

## Introduction

The incidence of epilepsy is highest in the first year of life with a reported rate around 1.8–3.5/1000 live births in the United States [1]. Although many antiepileptic drugs (AEDs) have been developed for the treatment of adult epilepsy over the past several decades, neonatal seizures still lack safe and effective treatment [2, 3]. In some cases, even after AED application, electroencephalographic (EEG) recordings still show ongoing cortical epileptic activity in neonates, which may impair cognitive development and later result in epilepsy [2, 4, 5]. Unfortunately, to date there have been few drugs that can effectively treat neonatal seizures, prompting an urgent need to search for new drugs.

Epileptic seizures are often caused by over-excitation of neural circuits. Because GABA_A_ receptors (GABA_A_-Rs) are the major inhibitory receptors in the adult brain, AEDs are often developed to increase GABA_A_-Rs function, such as benzodiazepine and barbiturate drugs [6]. However, while GABA_A_-Rs are mostly inhibitory in the adult brain, they can be excitatory in developing brains [7, 8]. Therefore, many AEDs that boost GABA function are often ineffective in controlling neonatal seizures, and sometimes even exacerbate neonatal seizure activity [9, 10]. Classically, GABA excitatory versus inhibitory function has been attributed to the regulation by Cl^−^ co-transporters NKCC1 and KCC2 [8, 11]. A previous study suggested that NKCC1 might be a potential drug target for the treatment of neonatal seizures [12], but recent clinical trials in human infants found severe side effect of NKCC1 blocker bumetanide and limited effect in treating neonatal seizure [13]. Because GABAergic transmission plays an important role in brain development [8, 11], blocking NKCC1 may potentially alter normal brain functions [14, 15]. Besides Cl^−^ co-transporters, Cl^−^ channels [16–18] and impermeant anions [19] also contribute to the regulation of Cl^−^ homeostasis and affect the epileptiform activity in the brain. Interestingly, the CLC-3 knockout mice show spontaneous generalized tonic-clonic seizures in adult animals, but not young animals (4–5 weeks) [20]. Another study reported a slight down-regulation of CLC-3c chloride channels during development [21]. It is unclear whether these two studies have any relationship. More broadly, whether Cl^−^ channels are involved in neonatal epilepsy is largely unknown.

Here, we report that voltage-dependent CLC-3 Cl^−^ channels play an important role in neonatal epileptiform activity. We first demonstrated in cultured neurons that gluconate inhibited voltage-dependent Cl^−^ currents and epileptiform activities. We then found in brain slices that CLC-3 Cl^−^ channels mediated a large voltage-dependent outward rectifying Cl^−^ current in neonatal but not in adult mouse brains. Gluconate effectively suppressed epileptiform activity in neonatal brain slices but had a lesser effect in adult brain slices. We further demonstrated with in vivo EEG recordings that gluconate was more effective in inhibiting seizure activity in neonatal animals than in adult animals. Finally, we demonstrated that activation of CLC-3 Cl^−^ channels during epileptiform activity significantly increased intracellular Cl^−^ concentration ([Cl^−^]_i_) and enhanced GABA excitation, whereas knocking out or blocking CLC-3 Cl^−^ channels with gluconate reduced [Cl^−^]_i_ and suppressed over-excitation of GABA. Thus, gluconate inhibits neonatal seizure activity through blocking CLC-3 Cl^−^ channels.

## Results

### Gluconate effectively inhibits epileptiform activity in cultured neurons

**Fig. 1:**
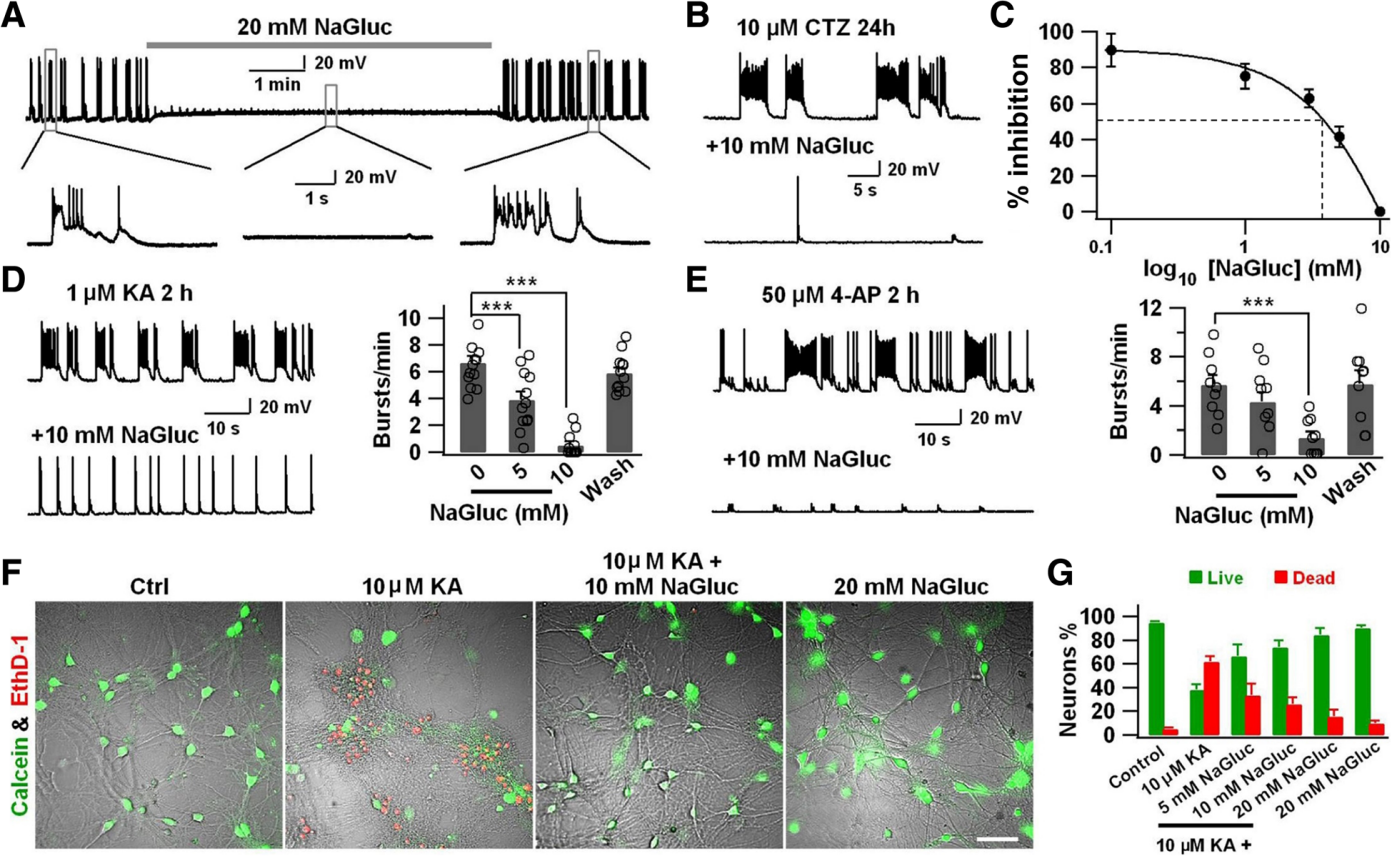
Gluconate inhibits epileptiform burst activity in cultured neurons. **a** Inhibition of spontaneous burst activity by 20 mM NaGluc in cultured cortical neurons (*n* = 10, from 3 batches). **b** CTZ-induced robust epileptiform activity (top trace) was completely blocked by 10 mM NaGluc (bottom trace). **c** Dose-dependent inhibition of NaGluc on CTZ-induced burst activity (*n* = 11 from 4 batches). **d** Kainic acid (KA, 1 μM for 2 h) induced epileptiform burst activity (top trace), and its suppression by 10 mM NaGluc (bottom trace). Bar graph showing the dose response (*n* = 12 from 3 batches, paired Student’s *t*-test). **e** Typical epileptiform activity induced by 4-AP (50 μM for 2 h) (top trace), and its inhibition by 10 mM NaGluc (bottom trace). Bar graph showing the dose response (*n* = 9 from 3 batches, paired Student’s *t*-test)**. f** Live/dead cell assay showing neuroprotective role of NaGluc against KA-induced cell death. Scale bar, 100 μm. **g** Quantification of cell survival under KA or KA plus NaGluc treatment. Note that NaGluc reduced cell death induced by KA, but itself had no side effect on neuron survival. Data are presented as mean ± s.e.m. *** *P* < 0.001

The functional roles of cation channels such as Na^+^, K^+^, and Ca^2+^ channels in epilepsy have been well documented in previous studies [22–24], but the function of anion channels in epilepsy is not well understood. Here, we report a serendipitous finding of gluconate inhibition of both Cl^−^ channels and epileptiform activity, leading to a new revelation of the relationship between Cl^−^ channels and epilepsy. We initially investigated how external Cl^−^ in bath solution regulates neuronal activity in cortical cultures. To our surprise, when we replaced only 20 mM NaCl with 20 mM sodium gluconate (NaGluc, Sigma G9005) in the bath solution, which contained a total of 139 mM Cl^−^, the spontaneous burst activity was effectively blocked (Fig. 1a). This was an unexpected finding because a mere difference of 20 mM Cl^−^, from 139 mM to 119 mM, is unlikely to explain why all burst activity had been inhibited. We therefore hypothesized that such strong inhibition could be caused by gluconate itself, rather than by the minor change of extracellular Cl^−^. To test this hypothesis, we examined the effect of gluconate on the robust epileptiform activity induced by a convulsant drug cyclothiazide (CTZ) [25]. Indeed, we found that CTZ-induced epileptiform activity was significantly blocked by application of 10 mM NaGluc in the bath solution (Fig. 1b-c). Furthermore, we found that gluconate also significantly inhibited the epileptiform activities induced by kainic acid (KA) (Fig. 1d) and 4-AP (Fig. 1e). Together, these data indicate that NaGluc exerts a strong inhibitory effect on epileptiform burst activity in cultured neurons.

### Gluconate protects cultured neurons against KA-induced cell death

Recurrent epileptic activity may induce cell death both in epileptic patients and animal models [26]. To evaluate whether the anti-epileptic effect of NaGluc might be neuroprotective, we employed kainic acid (KA) induced cell death model [25], and used the cytotoxicity kit (Life Technologies, L3224) to analyze the neuronal survival rate after KA treatment. In the control group, most neurons appeared to be healthy and stained by calcein (green, live cell marker) (Fig. 1f, left). After exposure to 10 μM KA for 24 h, most neurons were dead as shown by staining of cell death marker ethidium homodimer-1 (EthD-1, red) (Fig. 1f, middle left). Interestingly, co-application of 20 mM NaGluc with KA greatly reduced neuronal death (Fig. 1f, middle right). As a control, neurons exposed to 20 mM NaGluc alone had no detriment to cell survival (Fig. 1f, right). Quantitative analysis showed a dose-dependent neuroprotective effect of NaGluc (Fig. 1g). These experiments suggest that gluconate protects cultured neurons against KA-induced excitotoxicity.

### Gluconate inhibits Cl^−^ channels

**Fig. 2:**
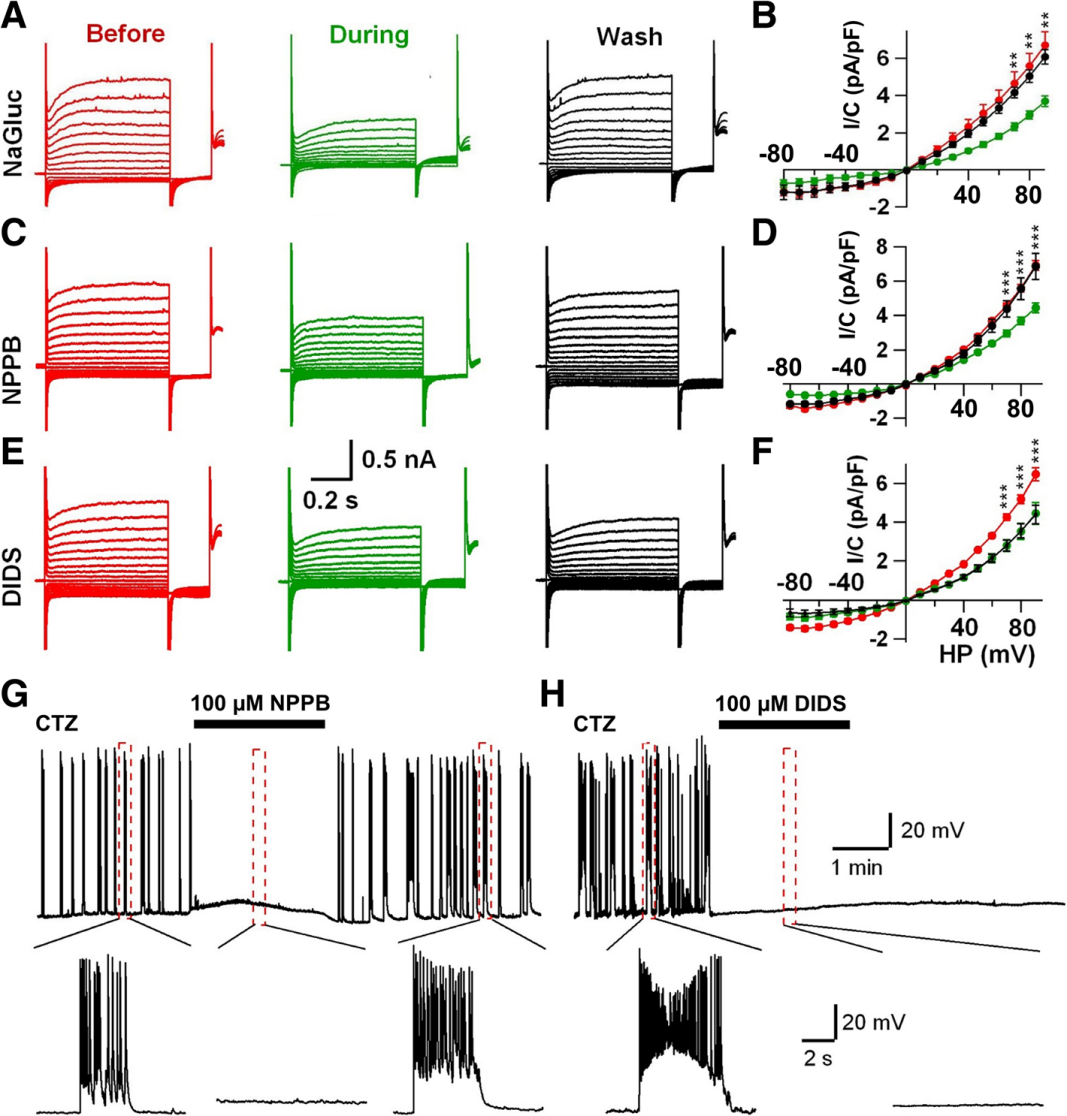
Gluconate inhibits Cl^−^ current in cultured neurons. **a, b** Typical Cl^−^ currents recorded before (red), during 10 mM NaGluc application (green), and wash out NaGluc (black). I-V curves showing a significant inhibition of NaGluc on the Cl^−^ currents (*n* = 7 from 3 batches of cultures, paired Student’s *t*-test). **c-f** NPPB and DIDS (100 μM), two classical Cl^−^ channel blockers, inhibited the Cl^−^ currents in cultured neurons. **g, h** Both NPPB (**g**) and DIDS (**h**) inhibited the epileptiform activity induced by CTZ. Data are presented as mean ± s.e.m. ** *P* < 0.01, *** *P* < 0.001

We next investigated why NaGluc can have such an inhibitory effect on epileptiform activity. Because gluconate ion is an organic anion used to replace Cl^−^, we investigated its potential effect on anion and cation channels. In the presence of NaGluc (10 mM), we found no significant changes in Na^+^, K^+^ and Ca^2+^ currents in neuronal cultures (Additional file 1: Figure S1A-F). Note that, gluconate might chelate Ca^2+^ in alkaline condition but not in physiological pH range (7.2–7.4). We found no effect of gluconate on Ca^2+^ currents in cell cultures or brain slices (Additional file 1: Figure S1G, H). Thus, it must be a mechanism other than Ca^2+^ current change underlying the gluconate inhibition of the burst activity. Indeed, we found that the voltage-dependent Cl^−^ currents were significantly decreased in the presence of 10 mM NaGluc (Fig. 2a, b; control, 913 ± 171 pA; NaGluc, 499 ± 89 pA; *n* = 7; *P* < 0.007, paired Student’s *t*-test; holding potential at + 90 mV). Thus, NaGluc may act as a Cl^−^ channel blocker, which is consistent with previous report that gluconate can block Cl^−^ channels in glioma cells [27]. Since NaGluc also inhibited epileptiform activity, we wondered whether these results indicated a potential link between Cl^−^ channels and epileptiform activity. To test this hypothesis, we examined two widely used Cl^−^ channel blockers, 5-Nitro-2-(3-phenylpropylamino) benzoic acid (NPPB) and 4,4′-Diisothiocyanato-2,2′-stilbenedisulfonic acid disodium salt (DIDS). As expected, both NPPB (100 μM) and DIDS (100 μM) suppressed Cl^−^ currents in cultured neurons (Fig. 2c-f). Interestingly, they also inhibited the epileptiform activity induced by CTZ (Fig. 2g, h, *n* = 9). Together, these results suggest that Cl^−^ channels are closely linked to epileptiform activity.

Compared to other Cl^−^ channel blockers such as NPPB and DIDS, gluconate has been widely used as a food additive or a drug additive approved by the FDA. Therefore, we decided to focus our study on gluconate in the rest of our experiments to further test whether gluconate can inhibit epileptiform activity in brain slices or live animals.

### Downregulation of CLC-3 Cl^−^ channels during brain development

**Fig. 3:**
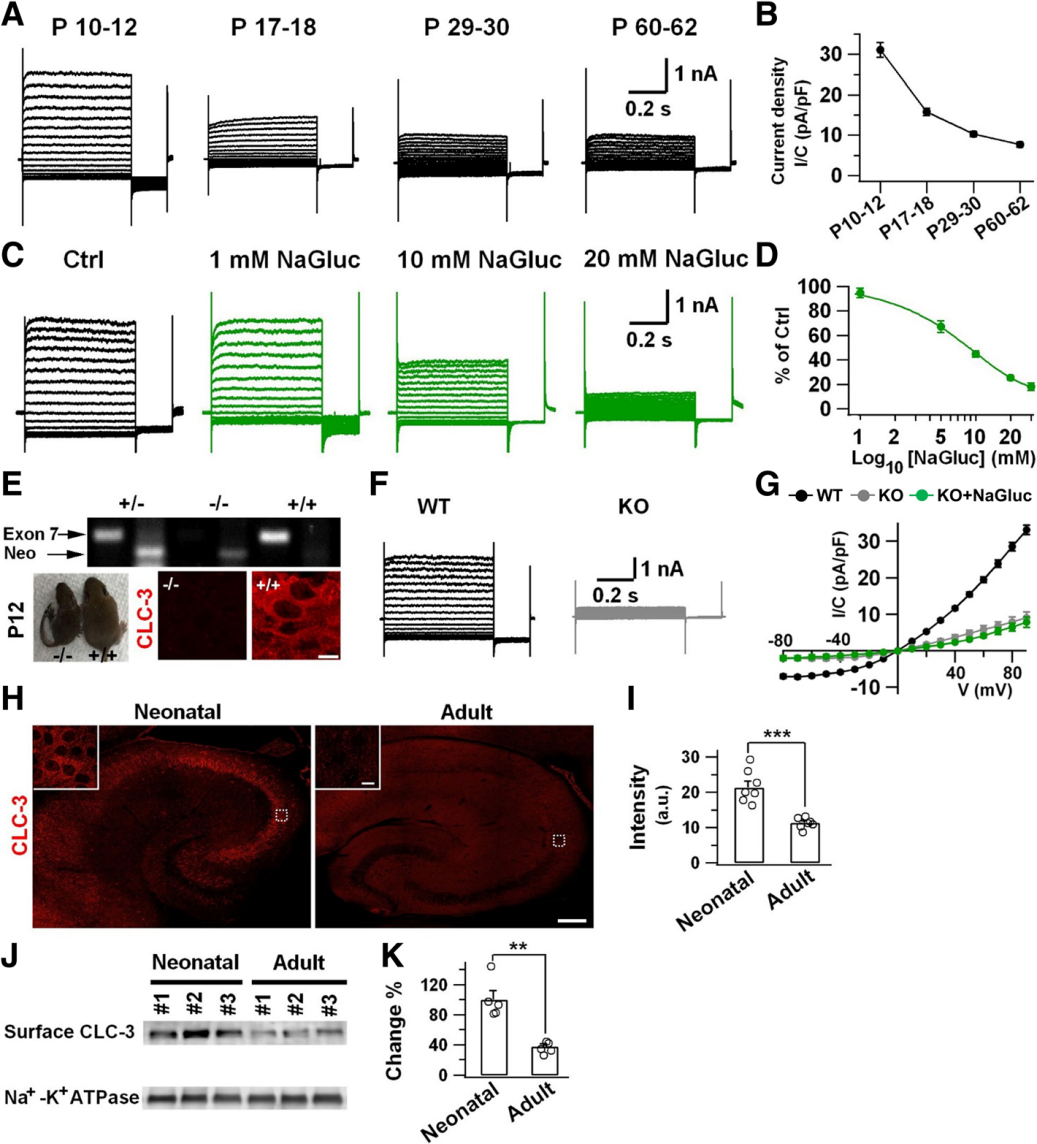
Developmental change of CLC-3 Cl^−^ channels in the mouse brain. **a** Representative traces of voltage-dependent Cl^−^ currents recorded in CA3 pyramidal neurons at different ages of animals. **b** Quantified Cl^−^ current density (HP = + 90 mV) illustrating age-dependent decline during early brain development. **c** NaGluc inhibition of Cl^−^ currents in WT neonatal neurons (P10–12). **d** Dose-response curve of NaGluc inhibition on Cl^−^ currents in hippocampal slices. **e** Characterization of CLC-3 knockout mice. PCR analysis confirmed a lack of the exon 7 of *Clcn3* gene. Immunostaining also confirmed a lack of CLC-3 expression in hippocampal CA3 region of the *Clcn3*^*−/−*^ mice. Note the smaller size of the *Clcn3*^*−/−*^ mice at P12 compared to WT mice. Scale bar = 10 μm. **f** Brain slice recordings revealed a voltage-dependent outward rectifying Cl^−^ current in WT hippocampal CA3 pyramidal neurons, but not in CLC-3 KO neurons (P8–12). **g** I-V plot of voltage-dependent Cl^−^ currents in WT (black) and CLC-3 KO (gray) neurons. The green curve shows no further inhibition of NaGluc on the remaining Cl^−^ currents in CLC-3 KO neurons. **h** Typical immuno-fluorescent images showing different expression level of CLC-3 Cl^−^ channels in the hippocampus of neonatal (P11, left panel) and adult mice (3.5 months, right panel). The high magnification images of CA3 were placed in the up-left corner. Low magnification image scale bar = 200 μm; Inset scale bar = 10 μm. **i** Quantified data showing CLC-3 immunostaining intensity in neonatal and adult CA3 regions (unpaired Student’s *t*-test, *P* < 0.001). **j, k** Western blot revealed a significant decrease of surface CLC-3 Cl^−^ channels in the adult hippocampus (Mann-Whitney test, *P* < 0.01). Data are shown as mean ± s.e.m., ** *P* < 0.01, *** *P* < 0.001

After the cell culture study, we further investigated the effect of gluconate on the Cl^−^ currents in hippocampal slices. To isolate Cl^−^ currents, the Na^+^, K^+^ and Ca^2+^ cations in the bath solution were all replaced by impermeable cation NMDG^+^ to reduce their potential effects. High Cl^−^ concentration in the pipette solution (140 mM Cl^−^) was used for the Cl^−^ current recording except where otherwise stated. Whole-cell patch-clamp recordings revealed a large voltage-dependent outward rectifying Cl^−^ current (3.3 ± 0.3 nA) in the CA3 pyramidal neurons of neonatal mice (P8–12) (Fig. 3a, top left panel). Surprisingly, the Cl^−^ current decreased significantly in the adult brains (P60–62) (Fig. 3a, b). Such dramatic downregulation of voltage-dependent outward rectifying Cl^−^ current during brain development was unexpected. Importantly, application of NaGluc significantly inhibited the Cl^−^ currents in neonatal hippocampal slices (Fig. 3c, d). The IC_50_ of NaGluc on the Cl^−^ currents in neonatal hippocampal neurons (P10–12) was about 8.6 mM (Fig. 3d). To ensure that the large Cl^−^ current was not caused by the high Cl^−^ concentration in the pipette solution, we recorded Cl^−^ currents using 15 mM Cl^−^ in the pipette solution (125 mM acetate in pipette solution), a concentration closer to physiological level, and found similar inhibition by NaGluc (Additional file 1: Figure S2). Thus, gluconate is an inhibitor of the voltage-dependent Cl^−^ channels in neonatal brains.

We next investigated the molecular identity of the voltage-dependent Cl^−^ currents. A previous study revealed a large CLC-3 Cl^−^ channel mediated outward rectifying current in hippocampal cultures [18]. Therefore, we investigated CLC-3 Cl^−^ channels in the brains of both wildtype (WT) and *Clcn3*^*−/−*^ mice. Knockout of CLC-3 was confirmed by PCR and immunohistochemistry, which also showed significantly smaller body size compared to the WT littermates (Fig. 3e). Whole-cell recordings from WT hippocampal CA3 pyramidal neurons showed a large voltage-dependent outward rectifying Cl^−^ current in neonatal brain slices (Fig. 3f, WT, P8–12), similar to that reported previously in cultured hippocampal neurons [18]. However, in neonatal *Clcn3*^*−/−*^ mice (P8–12, before hippocampal degeneration) [20, 28], the large Cl^−^ current was remarkably reduced (Fig. 3f, g; WT: 33.1 ± 1.3 pA/pF, + 90 mV, *n* = 10 vs CLC-3 KO: 9.0 ± 1.6 pA/pF, + 90 mV, *n* = 6. *P* < 0.001, unpaired Student’s *t*-test). Application of NaGluc to CLC-3 KO neurons had no further effect on the remaining small current (Fig. 3g). These results indicate that the Cl^−^ current in neonatal CA3 pyramidal neurons is mainly mediated by CLC-3 chloride channels.

To more directly test the idea of NaGluc as an inhibitor of CLC-3 Cl^−^ channels, we overexpressed CLC-3 Cl^−^ channels in HEK293T cells [29] and confirmed their expression with CLC-3 specific antibodies (Additional file 1: Figure S3A). Whole-cell recordings revealed large outward rectifying Cl^−^ currents in CLC-3-transfected HEK293T cells (1012 ± 123 pA, *n* = 7), but not in EGFP-transfected control cells (Additional file 1: Figure S3B). Application of 20 mM NaGluc significantly reduced the CLC-3 channel-mediated Cl^−^ currents (Additional file 1: Figure S3B, C), confirming previous report that NaGluc is an inhibitor of CLC-3 Cl^−^ channels [27].

We further investigated why adult mouse brains lacked the voltage-dependent outward rectifying Cl- currents. Using both immunostaining and Western blot analyses (Fig. 3h-k), we found a significant downregulation of CLC-3 Cl^−^ channels in the adult hippocampus, which is consistent with our electrophysiological results (Fig. 3a, b). Thus, CLC-3 Cl^−^ channels undergo a significant developmental change during brain development, a phenomenon also widely reported for other channels and receptors [30, 31].

### CLC-3 Cl^−^ channels contribute to recurrent ictal epileptiform activity in the developing hippocampus

**Fig. 4:**
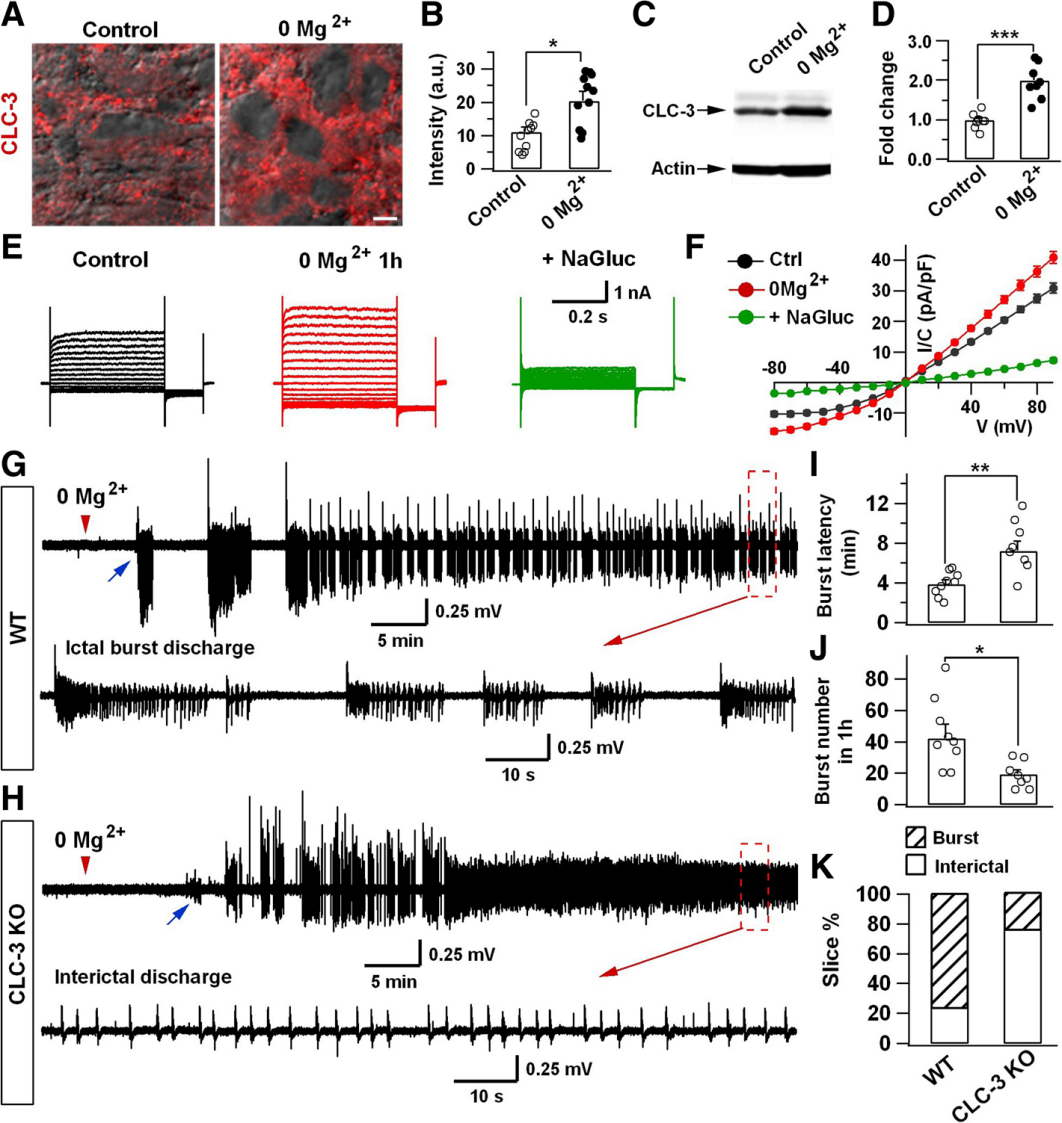
CLC-3 Cl^−^ channels and neonatal epileptiform activity. **a, b** Immunostaining illustrating an upregulation of CLC-3 Cl^−^ channel expression after 0 Mg^2+^ aCSF treatment (1 h). **c, d** Western blot analysis also confirmed an increase of CLC-3 Cl^−^ channel protein level after 0 Mg^2+^ treatment. Scale bar = 5 μm. **e** Representative voltage-dependent Cl^−^ current traces in control, 0 Mg^2+^ (1 h), and 0 Mg^2+^ + 20 mM NaGluc (1 h) conditions. **f** I-V curves showing a significant increase of outward rectifying Cl^−^ currents in 0 Mg^2+^ aCSF group (red) and a remarkable inhibition by NaGluc (green). **g, h** Representative traces of epileptiform activity induced by 0 Mg^2+^ aCSF in the hippocampal slices from WT (**g**) and CLC-3 KO mice (**h**) (P10–12). The blue arrowhead indicates the first ictal burst activity induced by 0 Mg^2+^ aCSF. Note that in the neonatal hippocampal slices from CLC-3 KO mice, the initial epileptiform ictal burst activity later transformed into interictal single spikes (**h**). **i, j** Summarized data showing the burst latency (**i**) and burst number (**j**) induced by 0 Mg^2+^ aCSF in hippocampal slices from WT and CLC-3 KO mice. **k** The percentage of slices showing ictal burst activity or interictal spike activity. Data are shown as mean ± s.e.m., unpaired Student’s *t*-test, *P < 0.05, ** *P* < 0.01, *** *P* < 0.001

Since we have discovered that NaGluc inhibited Cl^−^ currents and epileptiform activity in neuronal cultures, we wondered whether CLC-3 Cl^−^ channels identified in neonatal brain slices may regulate epileptiform activity. We first examined the expression level of CLC-3 Cl^−^ channels after induction of epileptiform activity by treating neonatal brain slices with 0 Mg^2+^ aCSF (artificial cerebral spinal fluid). Interestingly, epileptic stimulation significantly upregulated CLC-3 Cl^−^ channels in neonatal brain slices (Fig. 4a, b; control, 11.2 ± 1.5 a.u., *n* = 10; 0 Mg^2+^, 20.4 ± 2.8 a.u., *n* = 11; *P* < 0.02, Student’s *t*-test; P8-P12). This upregulation of CLC-3 Cl^−^ channels after epileptic stimulation was further confirmed by Western blot analysis (Fig. 4c, d). Consistent with these findings, whole-cell recordings revealed a significant increase of Cl^−^ currents after 0 Mg^2+^ treatment, which was also significantly inhibited by 20 mM NaGluc (Fig. 4e, f; control, 31.2 ± 1.8 pA/pF, *n* = 15; 0 Mg^2+^, 42.2 ± 2.4 pA/pF, *n* = 15, *P* < 0.001; NaGluc, 7.3 ± 0.6 pA/pF, *n* = 11, *P* < 0.001; one-way ANOVA followed with Tukey post hoc tests). These results suggest that CLC-3 Cl^−^ channels may be involved in regulation of neonatal epileptiform activity.

To further investigate the functional role of CLC-3 Cl^−^ channels in neonatal epileptiform activity, we examined whether lack of CLC-3 Cl^−^ channels in KO mice might have any effect on the induction of epileptiform activity in neonatal animals. Interestingly, while 0 Mg^2+^ treatment quickly induced epileptiform burst activity in the hippocampal slices from WT neonatal mice (Fig. 4g), there was a significant delay in the onset of epileptiform activity in neonatal hippocampal slices from CLC-3 KO mice (Fig. 4h). Moreover, under 0 Mg^2+^ treatment, WT hippocampal slices showed high frequency ictal bursts that lasted more than one hour during our continuous recordings (Fig. 4g, bottom enlarged trace), whereas the hippocampal slices from CLC-3 KO mice only showed transient ictal bursts and then turned into interictal single spikes (Fig. 4h, bottom enlarged trace). Quantitatively, the latency to the onset of ictal burst activity increased by 86.2% in the CLC-3 KO slices compared to the control WT slices (Fig. 4i); and after 0 Mg^2+^ treatment (1 h), while 77.8% of WT slices (*n* = 9) showed continuous ictal burst activity, the CLC-3 KO slices displayed mainly interictal single spikes (75%, *n* = 8, *P* < 0.01, χ^2^ test) after initial transient bursts (Fig. 4j, k). These results indicate that the absence of CLC-3 Cl^−^ channels may disrupt the sustainability of ictal epileptiform bursts.

### Inhibition of CLC-3 Cl^−^ channels suppresses epileptiform activity in the developing hippocampus

**Fig. 5:**
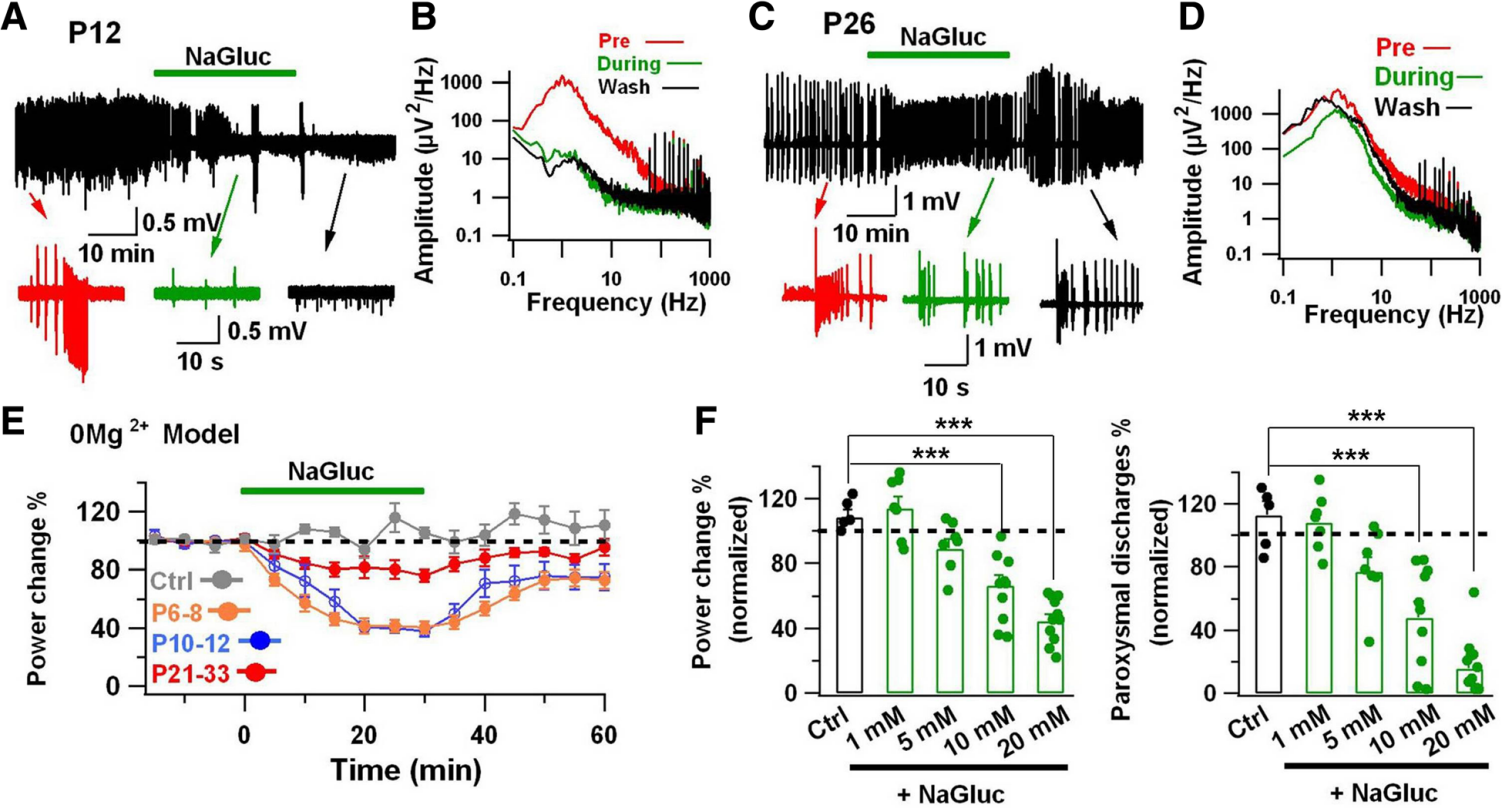
Suppressing neonatal epileptiform activity through inhibiting CLC-3 channels. **a** Field potential recording showing strong inhibition of epileptiform activity (0 Mg^2+^) by NaGluc (20 mM) in the CA3 pyramidal layer of hippocampal slices from neonatal mice (P12). **b** Power spectra of epileptiform activity (5-min time windows) before (red), during (green), and after (black) NaGluc application. The amplitude of power (integrative area under the power spectrum trace) was significantly reduced after NaGluc application. **c, d** In P26 hippocampal slices, however, NaGluc only showed modest inhibitory effect on the epileptiform activity. **e** Normalized power showing the time course of NaGluc inhibition on the epileptiform activity induced by 0 Mg^2+^ aCSF. NaGluc dramatically reduced the power of epileptiform activity in the P6–8 and P10–12 neonatal slices (*P* < 0.001, paired Student’s *t*-test). As a control, the gray line represents the effect of adding 20 mM NaCl on neonatal (P8–12) epileptiform activity, there was no significant change (*P* > 0.1, paired Student’s *t*-test). **f** Dose-dependent inhibition of NaGluc on the power (left) and paroxysmal discharges (right) in the neonatal brain slices. The percentage change of power and paroxysmal discharges were normalized to the 20 min of stable recordings before NaCl or NaGluc application. Control (Ctrl) represents the effect of 20 mM NaCl. Compared to control, the power and paroxysmal discharges were significantly reduced in the presence of 10 mM or 20 mM NaGluc. Data are shown as mean ± s.e.m. *** *P* < 0.001

The reduced epileptiform activity in neonatal CLC-3 KO mice prompted us to test whether inhibiting CLC-3 Cl^−^ channels by NaGluc would have any effect on epileptiform activity in neonatal brain slices. Interestingly, we observed a significant age-dependent difference in the effect of NaGluc on the epileptiform activity induced by 0 Mg^2+^ treatment. Specifically, NaGluc (20 mM) strongly inhibited the epileptiform activity in neonatal hippocampal slices (P7-P12) (Fig. 5a, b). However, in ~ 1-month old animals (P26), the inhibitory effect of NaGluc on epileptiform activity became much smaller (Fig. 5c, d). Quantitatively, NaGluc (20 mM) inhibited 60% of the average power of epileptiform activity in early postnatal animals (P6–8, 59.6 ± 4.3%, *n* = 10; P10–12, 62.1 ± 3.8%, *n* = 10; *P* < 0.001, paired Student’s *t*-test), but only reduced 20% of the power in older animals after weaning (P21–33, 23.8 ± 4.1%, *n* = 7; *P* < 0.005, paired Student’s *t*-test) (Fig. 5e). Moreover, gluconate inhibition of epileptiform activity in neonatal slices showed a clear dose-dependence (Fig. 5f). To exclude the effects of osmolarity change by adding NaGluc (2 ml of 1 M NaGluc stock solution), we also added 2 ml of 1 M NaCl stock solution into the 0 Mg^2+^ aCSF, and found that the neonatal epileptiform activity was not significantly changed (Fig. 5e, Ctrl). The age differences in the NaGluc inhibition of epileptiform activity coincides well with the different amplitudes of Cl^−^ currents observed in neonatal and adult brain slices (Fig. 3a, b), further supporting a close link between CLC-3 Cl^−^ channels and neonatal epileptiform activity.

Besides 0 Mg^2+^ stimulation, we further examined whether NaGluc could inhibit neonatal epileptiform activity induced by other hyperexcitatory stimulation. We first tested 4-AP model by adding K^+^ channel blocker 4-AP (50 μM) into the 0 Mg^2+^ aCSF to induce robust epileptiform activity (Additional file 1: Figure S4A). Addition of 20 mM NaGluc to the 4-AP + 0 Mg^2+^ aCSF significantly reduced the epileptiform activity in neonatal hippocampal slices (Additional file 1: Figure S4A-C; 70.2 ± 5.3% reduction of the power amplitude by NaGluc, *n* = 9; *P* < 0.001, paired Student’s *t*-test). The second test was on a high K^+^ model, where elevated extracellular K^+^ (8.5 mM) was used to induce epileptiform activity (Additional file 1: Figure S4D). Similarly, addition of 20 mM NaGluc into the high K^+^ aCSF dramatically reduced the epileptiform burst activity in neonatal hippocampal slices (Additional file 1: Figure S4D-F, NaGluc reduced the power amplitude by 70.8 ± 4.9%, *n* = 5; *P* < 0.001, paired Student’s *t*-test). In summary, our results demonstrate that gluconate is an anti-epileptiform activity agent in the neonatal animals.

We further identified that β-HB, a ketone body generated in the liver under ketogenic diet, is an inhibitor of CLC-3 chloride channels. Ketone bodies (acetoacetate and β-HB, purchased from TCI) are produced in the liver, released into the circulatory system, transported into the brain through monocarboxylate transporter, and then serve as the substrates for energy production in the brain but later also found as anti-epileptic agents [32, 33]. Ketone bodies and gluconic acid are both organic acids. β-HB and gluconate, in particular, have similar structures and are both hydroxyl-containing monocarboxylates. Thus, we hypothesized that β-HB might be another inhibitor for CLC-3 chloride channels. We found that Cl^−^ current was indeed inhibited by β-HB but not acetoacetate (Additional file 1: Figure S5A). The IC_50_ of β-HB on the Cl^−^ currents in neonatal hippocampal neurons was about 6.2 mM (Additional file 1: Figure S5B). When we replaced glucose with β-HB in the recording medium, the epileptiform activity was significantly suppressed (Additional file 1: Figure S5C, D). The average power of epileptiform activity was decreased by 44.5% ± 3.2% (Additional file 1: Figure S5E, *n* = 7). These data suggest that the CLC-3 Cl^−^ channel might be a potential target of the ketone bodies.

### Gluconate inhibits neonatal seizure activity in vivo

**Fig. 6:**
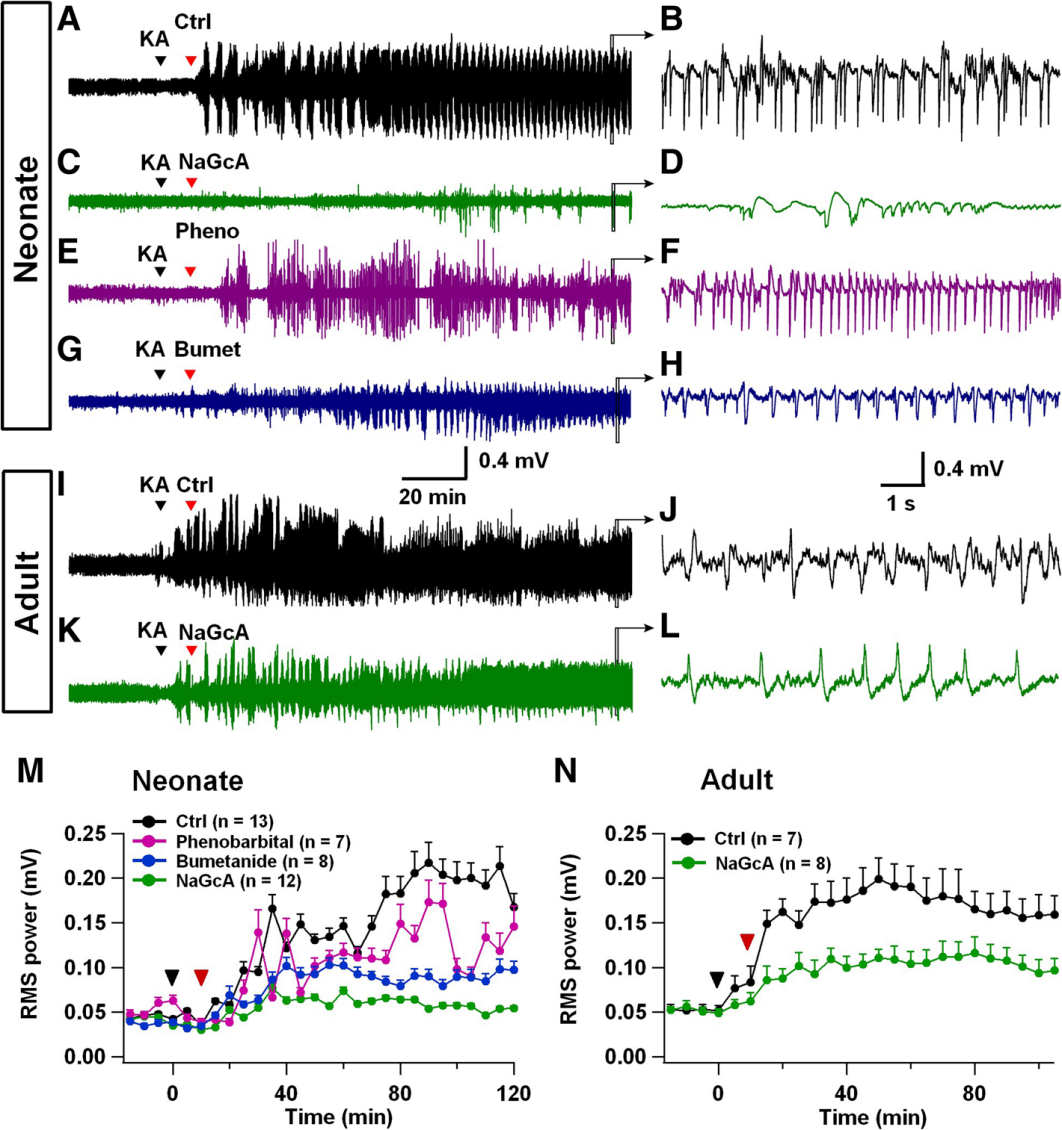
CLC-3 channel blocker gluconate potently inhibits neonatal seizure activity in vivo. **a** Representative EEG trace showing recurrent epileptic burst discharges from a P12 rat after KA injection (2 mg/kg, i.p.), which was followed by saline injection (0.1 ml/10 g, i.p.) with 10 min interval. **b** Expanded view of epileptic burst discharges from the box in **a**. **c, d** Representative EEG trace (P12 rat) showing that NaGluc injection (2 g/kg, i.p.) at 10 min after KA injection (2 mg/kg, i.p.) significantly inhibited epileptic burst discharges. **e, f** Representative EEG trace showing a modest effect of phenobarbital (25 mg/kg, i.p.) on epileptic burst activities in neonatal rat (P12). **g, h** Representative EEG trace showing the effect of bumetanide (0.2 mg/kg, i.p.) on epileptic burst activities in neonatal rat (P12). **i-l** In adult mice, KA injection (10 mg/kg, i.p.) also induced robust epileptic burst activities as shown in EEG recordings (**i, j**), but NaGluc (2 g/kg, i.p.) only showed modest effect on KA-induced epileptic burst activities **(k, l)**. **m** Summarized data in neonatal animals showing the averaged EEG power (5-min time window) before and after KA injection, followed by injection of saline (black), NaGluc (green), phenobarbital (magenta), or bumetanide (blue). Note that NaGluc significantly inhibited the power of EEG increase induced by KA. Compared to control groups, the EEG power was reduced 72.4% by NaGluc (*P* < 0.01, unpaired Student’s *t*-test), 39.6% by phenobarbital (*P* < 0.04, unpaired Student’s *t*-test), and 52.8% by bumetanide (*P* < 0.04, unpaired Student’s *t*-test) for the last 20 min of drug administration. **n** In adult animals, the average power in the last 20 min was reduced 36.2% by NaGluc (*P* < 0.03, unpaired Student’s *t*-test). In both panels **m** and **n**, the black arrowhead indicates the KA injection, and the red arrowhead indicates drug injection. Data are mean ± s.e.m

Following brain slice work, we further investigated the effect of gluconate on in vivo seizure activity in neonatal and adult animals. We employed a commonly used seizure model induced by a neurotoxin kainic acid (KA) [12]. Because neonatal mice were too small for in vivo EEG recordings, we injected KA (2 mg/kg, i.p.) into neonatal rats (P10–12) to elicit robust seizure activities as revealed by EEG recordings (Fig. 6a, b) [12]. Importantly, when NaGluc (2 g/kg, i.p.) was injected 10 min after KA injection, the epileptic seizure activity was inhibited in neonatal animals (Fig. 6c, d). Furthermore, we compared the anti-epileptic effect of gluconate with previously reported anti-convulsant drugs such as phenobarbital and bumetanide in neonatal animals. Phenobarbital is currently the drug of first choice to treat neonatal seizures, despite only ~ 50% efficacy and potential negative neurodevelopmental consequences [34]. Bumetanide is a loop diuretic, currently under evaluation as a potential antiepileptic drug [10]. Both phenobarbital (25 mg/kg, i.p.) and bumetanide (0.2 mg/kg, i.p.) also inhibited seizure activity to a certain degree in neonatal animals (Fig. 6e-h). Quantitatively, when we calculated the EEG power in the last 30 min during our 2-h recording period after KA injection, we found that the relative power was reduced by 72.3% in NaGluc group, 35.5% in phenobarbital group, and 54.3% in bumetanide group, respectively (Fig. 6m). We further analyzed the anti-epileptic effect of gluconate in adult animals. Compared to the significant inhibition of neonatal seizure, gluconate showed less inhibition of adult seizure activity (Fig. 6i-l and n), consistent with our results in brain slice recordings (Fig. 5a-e). Therefore, we conclude that NaGluc may be a new anti-epileptic drug to treat neonatal seizures.

Besides KA-induced neonatal seizure model, we further investigated the effect of gluconate on a more clinically relevant neonatal seizure model induced by hypoxia-ischemia (HI) stimulation [35, 36]. Combining ischemia (right common carotid artery ligation) with hypoxia (10% O_2_ for 2 h) stimulation (see Additional file 1: Figure S6 for experimental illustration), we were able to observe clear epileptic seizure activity through EEG recordings in neonatal rats (Additional file 1: Figure S7A and B). Importantly, application of NaGluc (2 g/kg, i.p.) significantly reduced the HI-induced seizure activity (Additional file 1: Figure S7C and D). Similarly, phenobarbital (25 mg/kg, i.p.) also inhibited the HI-induced seizure activity in neonatal animals (Additional file 1: Figure S7E and F). Unexpectedly, addition of gluconate and phenobarbital together even more strongly suppressed the seizure activity (Additional file 1: Figure S7G and H), suggesting a potential synergistic effect between these two drugs. Quantitatively, both the seizure burst number and burst duration were significantly reduced by NaGluc or phenobarbital, and no bursts were detected in the presence of both drugs (Additional file 1: Figure S7I and J). Therefore, combining NaGluc together with phenobarbital may yield a novel treatment for neonatal seizures.

**Fig. 7:**
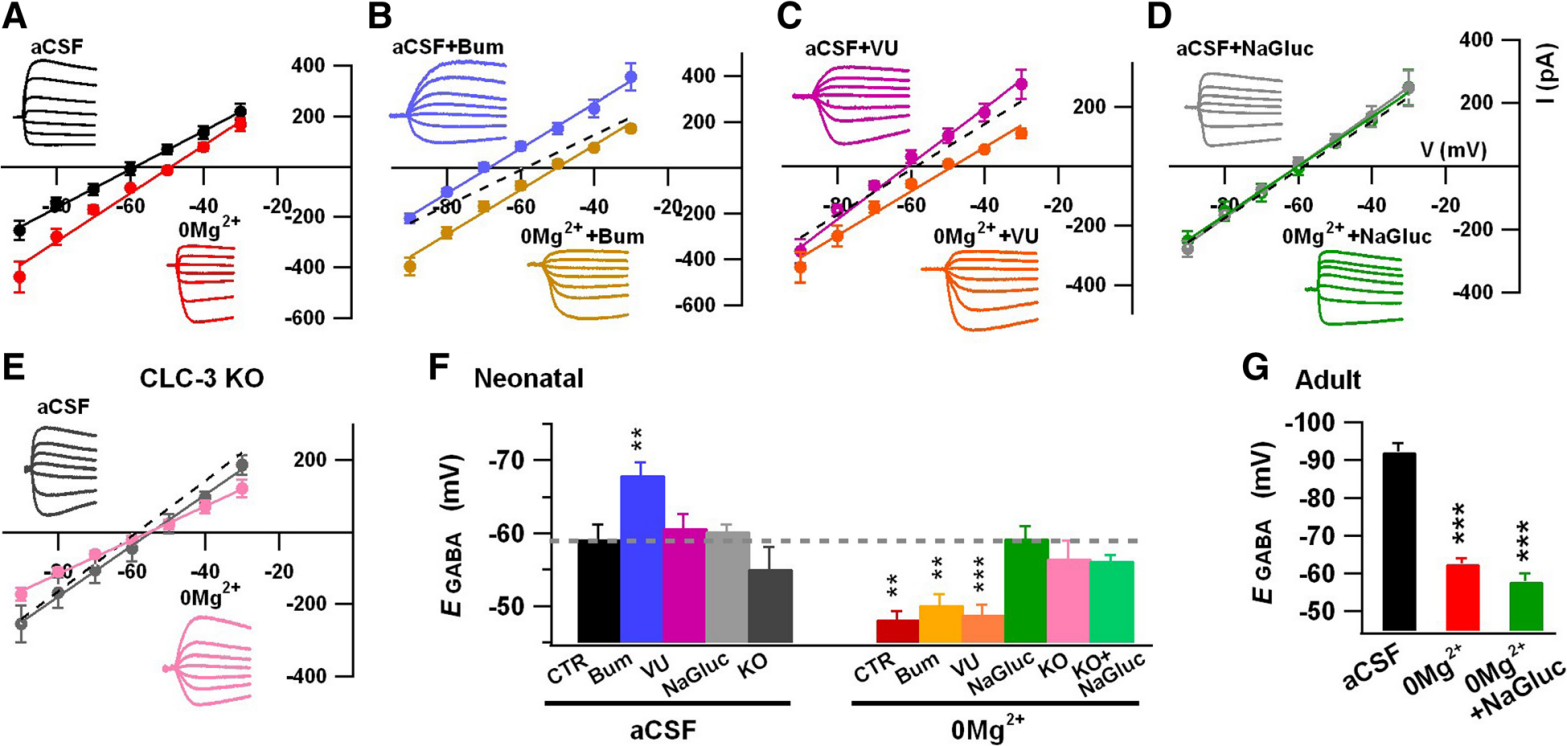
CLC-3 Cl^−^ channels regulate *E*_GABA_ during epileptic stimulation in neonatal neurons. **a** Gramicidin-perforated recordings revealed a depolarizing shift in the GABA_A_-R reversal potential (*E*_GABA_) in CA3 neurons after induction of epileptiform activity in 0 Mg^2+^ aCSF. **b** Bath application of NKCC1 inhibitor bumetanide induced a hyperpolarizing shift in *E*_GABA_ in normal aCSF, but did not abolish the depolarizing shift of *E*_GABA_ induced by 0 Mg^2+^ aCSF. **c** KCC2 inhibitor VU0240551 had no effect on the *E*_GABA_ under normal aCSF, and showed no effect on the depolarizing shift induced by 0 Mg^2+^ aCSF. **d** Gluconate strongly inhibited the depolarizing *E*_GABA_ shift induced by 0 Mg^2+^ aCSF. Gluconate itself did not affect the *E*_GABA_ in normal aCSF. **e** In CLC-3 KO mice, *E*_GABA_ did not change when treated with 0 Mg^2+^ aCSF. The dash line in panels **b-e** shows the control I-V plot in normal aCSF in **A** (black line). (**F**) Quantified data showing the *E*_GABA_ changes under various conditions in neonatal CA3 pyramidal neurons (P8–9). **g** Bar graphs showing *E*_GABA_ changes in adult CA3 pyramidal neurons. Note that NaGluc (20 mM) could not abolish the *E*_GABA_ shift induced by 0 Mg^2+^ aCSF in adult animals. Data are shown as mean ± s.e.m. ** *P* < 0.01, *** *P* < 0.001

### CLC-3 Cl^−^ channels regulate Cl^−^ homeostasis in neonatal neurons

Since knocking out or inhibiting CLC-3 Cl^−^ channels significantly suppresses the neonatal epileptiform activity, we investigated the underlying mechanism underlying the CLC-3 Cl^−^ channels and epileptiform activity. Because CLC-3 Cl^−^ channel is a voltage-dependent outward rectifying Cl^−^ channel, we hypothesize that CLC-3 Cl^−^ channels may regulate Cl^−^ homeostasis, which will in turn affect GABA function and recurrent ictal burst epileptiform activity. Using gramicidin-perforated whole-cell recordings to keep [Cl^−^]_i_ intact, we found that epileptic stimulation with 0 Mg^2+^ aCSF (32 °C for 1 h) induced a depolarizing shift in *E*_GABA_ in neonatal hippocampal CA3 pyramidal neurons (P8–9, aCSF: − 59.2 ± 2.0 mV, *n* = 13; 0 Mg^2+^: − 48.2 ± 1.1 mV, *n* = 10) (Fig. 7a). Treatment of neonatal slices with bumetanide (Bum, 10 μM), a specific blocker for NKCC1 at low concentration, induced a hyperpolarizing shift in *E*_GABA_ in the normal condition (Fig. 7b, blue line, dashed line indicates control *E*_GABA_), because NKCC1 imports Cl^−^ into neuronal cells [10–12]. However, in the presence of bumetanide, epileptic stimulation still elicited a depolarizing shift of *E*_GABA_, suggesting that a factor other than NKCC1 is regulating *E*_GABA_ during epileptic stimulation (Fig. 7b, yellow line; Bum, − 68.0 ± 1.7 mV, *n* = 9; 0 Mg^2+^ + Bum, − 50.2 ± 1.4 mV, *n* = 9; *P* < 0.002). Treatment with KCC2 blocker VU0240551 (10 μM) did not affect the *E*_GABA_ in neonatal animals (Fig. 7c, purple line; − 60.8 ± 1.9 mV, *n* = 6), possibly due to a low expression level of KCC2 at this early time [8, 11, 12]; and epileptic stimulation still elicited a positive shift in *E*_GABA_ (Fig. 7c, orange line; − 48.9 ± 1.3 mV, *n* = 8). Therefore, the *E*_GABA_ shift induced by epileptic stimulation in neonatal CA3 pyramidal neurons is not controlled by NKCC1 or KCC2.

Then, we asked whether CLC-3 Cl^−^ channels are involved in the *E*_GABA_ shift induced by epileptic stimulation. Interestingly, when we inhibited CLC-3 Cl^−^ channels with NaGluc (20 mM), the *E*_GABA_ was not altered by epileptic stimulation (Fig. 7d; 0 Mg^2+^ + NaGluc: − 59.3 ± 1.7 mV, *n* = 6). Furthermore, in CLC-3 KO mice, the *E*_GABA_ also remained unchanged by epileptic stimulation (Fig. 7e; CLC-3 KO + 0 Mg^2+^: − 55.6 ± 2.1 mV, *n* = 10), and application of NaGluc to CLC-3 KO neurons had no additive effect on *E*_GABA_ (− 56.6 ± 2.4 mV, *n* = 5). Importantly, inhibiting CLC-3 channels with NaGluc or knockout of CLC-3 channels did not change the *E*_GABA_ in normal conditions (Fig. 7f; NaGluc: − 60.3 ± 0.9 mV, *n* = 5; CLC-3 KO: -55.4 ± 2.9 mV, *n* = 9)_._ In older animals (P30–90), however, NaGluc showed no effect on the *E*_GABA_ shift induced by epileptic stimulation (Fig. 7g), consistent with our observation that CLC-3 channel-mediated Cl^−^ current is greatly reduced in the adult animals (Fig. 3a, b). Together, our results suggest that CLC-3 Cl^−^ channels play a critical role in controlling *E*_GABA_ during epileptic stimulation in neonatal animals.

### CLC-3 Cl^−^ channels modulate GABA function

**Fig. 8:**
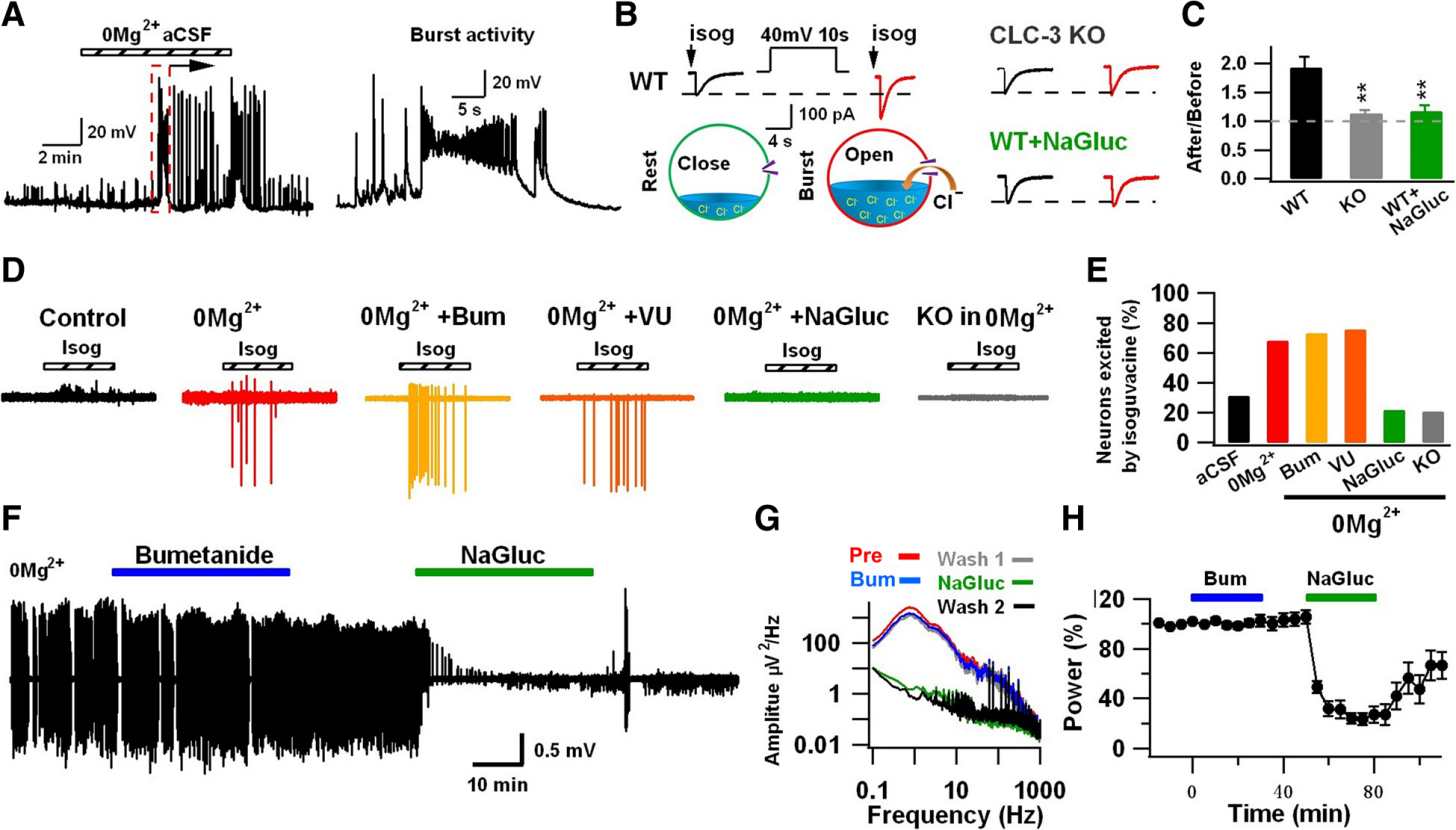
CLC-3 activation regulates GABA excitation in the developing neurons. **a** Epileptiform burst activity induced by 0 Mg^2+^ aCSF in neonatal CA3 neurons showed long-lasting membrane depolarization. **b** A long-lasting membrane depolarization pulse (40 mV, 10 s), mimicking the epileptiform burst activity, which significantly enhanced the inward GABA_A_-R current (actually Cl^−^ efflux trough the GABA_A_-R) induced by GABA_A_-R agonist isoguvacine (100 μM) under gramicidin-perforated whole-cell recording. Such depolarization-induced enhancement was absent in CLC-3 KO mice and strongly inhibited by CLC-3 channel blocker NaGluc (20 mM). **c** Summarized data of the long-lasting membrane depolarization effect on the inward GABA_A_-R current in WT, CLC-3 KO, and WT + NaGluc groups. ** *P* < 0.01. **d** Typical traces of cell-attached recording showing the spike activity induced by isoguvacine (10 μM, 30 s) under different conditions in neonatal animals (P8–9). **e** Summarized data showing the percentage of neurons excited by isoguvacine. Note that CLC-3 KO and NaGluc significantly inhibited the GABA excitatory activity induced by 0 Mg^2+^ aCSF. **f** Comparison between the effects of bumetanide and NaGluc on epileptiform activity induced by 0 Mg^2+^ in neonatal brain slices. **g, h** Power analysis illustrated a significant reduction of power after NaGluc application (68.9% reduction in presence of NaGluc, *P* < 0.001, paired Student’s *t*-test) but not bumetanide application (*P* > 0.7, paired Student’s *t*-test). Data are shown as mean ± s.e.m

Since GABAergic transmission plays an important role in recurrent seizure activity [37, 38], we wondered whether CLC-3 Cl^−^ channels might modulate GABA function in the developing brain. To test this idea, we investigated the effect of NaGluc on GABA_A_-R currents under conditions mimicking epileptic stimulation. We observed that 0 Mg^2+^ aCSF-induced epileptiform bursts often lasted more than 10 s in the neonatal CA3 pyramidal neuron (Fig. 8a). To investigate the effect of such long-lasting epileptiform bursts on GABA function, we performed gramicidin-perforated whole-cell recordings to keep the intracellular Cl^−^ intact in neonatal brain slices [7]. We recorded GABA_A_-R currents induced by isoguvacine (100 μM, 50 ms), an agonist for GABA_A_-Rs, before and after a membrane-depolarizing shift (40 mV for 10 s, holding potential was set at − 70 mV) that mimicked the epileptiform burst activity. Interestingly, the GABA_A_-R current (excitatory current, Cl^−^ efflux through the GABA_A_-Rs) was significantly increased after membrane depolarization in WT neurons but not in CLC-3 KO neurons, nor in the presence of CLC-3 channel blocker NaGluc (Fig. 8b, c). It appears that depolarizing stimulation activates CLC-3 Cl^−^ channels, and the Cl^−^ influx through CLC-3 Cl^−^ channels leads to intracellular accumulation of Cl^−^, which in turn enhances the GABA excitation (Fig. 8b, the working model).

To further test this hypothesis, we directly measured GABA-induced neuronal activity in neonatal brain slices (P8–9). Cell-attached recordings were performed to monitor neuronal firing activity elicited by local application of GABA_A_-R agonist isoguvacine (10 μM, 30 s) [39]. The majority of CA3 pyramidal neurons in the resting condition did not respond to isoguvacine (Fig. 8d, control, *n* = 32). However, after 0 Mg^2+^ treatment, 68% of neurons showed spike activity upon isoguvacine application (Fig. 8d, 0 Mg^2+^, *n* = 19). Blocking NKCC1 with bumetanide (Fig. 8d, 0 Mg^2+^ + Bum, *n* = 19) or blocking KCC2 with VU0240551 (Fig. 8d, 0 Mg^2+^ + VU, *n* = 21) did not change the percentage of neurons excited by isoguvacine. In contrast, knock out of CLC-3 or application of NaGluc to inhibit CLC-3 Cl^−^ channels significantly decreased the percentage of neurons excited by isoguvacine after 0 Mg^2+^ treatment (Fig. 8d, 0 Mg^2+^ + NaGluc, *n* = 18; CLC-3 KO, *n* = 19; quantified in Fig. 8e). These differential results of Bum and NaGluc on *E*_GABA_ and GABA activity suggest that NKCC1 Cl^−^ transporters and CLC-3 Cl^−^ channels may play different functions in the recurrent epileptiform activity in the neonatal brain.

We then directly compared the effects of NKCC1 Cl^−^ transporter inhibitor (Bum) versus CLC-3 Cl^−^ channel inhibitor (NaGluc) on neonatal epileptiform activity induced by Mg^2+^-free aCSF in neonatal hippocampal slices. After induction of epileptiform activity, we first applied Bum (10 μM) followed by NaGluc (20 mM). Blocking NKCC1 with Bum did not change the epileptiform activity induced by 0 Mg^2+^ aCSF (Fig. 8f-h), which is consistent with a previous study [40]. In contrast, application of NaGluc dramatically reduced the epileptiform activity in the same slices (Fig. 8f, g). Quantified data showed that the power was reduced ~ 70% during NaGluc application (Fig. 8h; *p* < 0.001, paired Student’s *t*-test, *n* = 8, P7-P9 pups), but no change was observed in bumetanide application (Fig. 8h). Therefore, we conclude that gluconate may inhibit neonatal seizure activity through blocking CLC-3 Cl^−^ channels and stabilizing intracellular Cl^−^ homeostasis.

## Discussion

In this study, we discovered that gluconate inhibits neonatal seizures through blocking CLC-3 Cl^−^ channels. Interestingly, CLC-3 Cl^−^ channels mediate a large voltage-dependent Cl^−^ current in neonatal brains, but not adult brains. Such developmental change of CLC-3 Cl^−^ channels coincides with a strong inhibition of gluconate on neonatal seizure activity but a moderate effect on adult seizure activity. Mechanistically, we demonstrate that CLC-3 Cl^−^ channels regulate Cl^−^ homeostasis and GABA function in neonatal neurons. Together, our studies demonstrate that gluconate suppresses neonatal seizure activity through the inhibition of CLC-3 Cl^−^ channels.

Previous studies have linked Cl^−^ channels in the CLC family to human epilepsy but the mechanism is not well understood. Mutations in CLC-1 channels have been identified in idiopathic epileptic patients [41]. CLC-2 channel mutations have also been found in human patients but some studies suggest that the mutations may not contribute to epilepsy [42–44]. CLC-3 channels are widely expressed in different brain areas, and the hippocampus is one of the highest expression regions [45, 46]. While the hippocampus is known to be one of the most epileptogenic structures in the brain, there has been little study directly investigating the relationship between CLC-3 channels and neonatal seizure, except a report that CLC-3 knock-out mice showed resistance to PTZ-induced seizure activity [20]. Our work provided a direct link between CLC-3 Cl^−^ channels and neonatal seizure.

CLC-3 chloride channels are members of an extended family of voltage-dependent chloride channels and transporters, with multiple functional properties and subcellular locations [28, 47]. The CLC-3 chloride channel is encoded by CLCN3 gene, which has 9 protein coding splice variants in the human genome and 5 in the mouse (Ensembl database). In 2015, it was found that the different splice variants of CLC-3 have different spatial distributions in the cell [21]. Interestingly, one of the neuronal CLC-3 splice variants, CLC-3c, prefers to locate at plasma membranes. After overexpressing in HEK293T cells, whole cell recordings revealed the outward rectifying Cl^−^ currents that were mediated by the CLC-3c channels. Meanwhile, they also found that the CLC-3c in the hippocampus was slightly down-regulated during development [21]. Consistent with this, we have confirmed that plasma membrane CLC-3 Cl^−^ channels are significantly down-regulated in the hippocampus during brain development, which may be due to a potential change in the CLC-3 splice variants reported recently [21]. A significant developmental change of CLC-3 Cl^−^ channels is reminiscent of many other channel and receptor changes during brain development [30, 31, 48]. CLC-3 channel-mediated Cl^−^ currents have also been recorded in cultured hippocampal neurons [18], which is consistent with our recordings in the hippocampal slices. In addition to neurons, CLC-3 channel-mediated Cl^−^ currents have also been detected in other types of cells [27, 49]. Our studies demonstrate that CLC-3 Cl^−^ channel-mediated currents regulate the intracellular Cl^−^ homeostasis, which in turn affects GABA function in the neonatal brains.

Plasma membrane depolarization will activate CLC-3 Cl^−^ channels, leading to a large amount of Cl^−^ influx that will significantly change the [Cl^−^]_in_. Therefore, in addition to NKCC1, CLC-3 also plays an important role in regulating intracellular Cl^−^ homeostasis in the developing brain. However, these transporters may play different roles due to their differences in voltage dependence. At rest, voltage-sensitive CLC-3 Cl^−^ channels are relatively silent, and therefore Cl^−^ transporters such as NKCC1 are the major players that maintain [Cl^−^]_i_ in developing neurons [8, 11, 50]. During epileptic stimulation, however, CLC-3 Cl^−^ channels are activated and the large Cl^−^ influx significantly increases [Cl^−^]_i_, resulting in GABA over-excitation and recurrent ictal seizure activity. In supporting this novel hypothesis, we demonstrate that neonatal CLC-3 knockout slices do not have large outward rectifying Cl^−^ current, and consequently the recurrent ictal burst activity is transformed to the interictal activity. Similarly, blocking CLC-3 Cl^−^ channels with gluconate strongly inhibits neonatal seizure bursts, possibly through disrupting the positive feedback loop between CLC-3 Cl^−^ channels and recurrent ictal burst activity. In conclusion, we have identified the CLC-3 Cl^−^ channel as a novel target for suppressing robust epileptiform activity in the developing brain and found that gluconate can inhibit neonatal epileptiform activity by blocking CLC-3 Cl^−^ channels. Since CLC-3 Cl^−^ channels also regulate NMDA receptors and GABAergic synaptic transmission [18, 28], gluconate may also suppress neonatal seizure activity through modulating NMDA receptors and GABAergic synaptic transmission. These questions deserve further study in the future.

Gluconic acid is a large organic anion, often used as a food or drug additive in a salt form such as magnesium gluconate, calcium gluconate, or potassium gluconate, where gluconate was used as an inactive ingredient to deliver cations. However, the functional role of gluconate itself was largely neglected in the past. One case study reported that an epileptic patient was treated with Ca-gluconate and then epileptic jerks faded [51]. The authors attributed this effect to Ca^2+^, but whether gluconate might have played any role was completely ignored. Notably, gluconic acid is a polyhydroxycarboxylic acid, with divalent cation chelating capability especially in alkaline solutions [52]. Since Ca^2+^ is very important for neurotransmission, the divalent cation chelation of gluconic acid might have an effect on the epileptiform activity. However, we demonstrate here that the Ca^2+^ current is not affected by bath application of gluconate under the physiologic pH (7.3–7.4), confirming that gluconate chelation depends on alkaline conditions. Moreover, we also directly compared the antiepileptic effect of gluconate between neonatal and adult rodents, and found a stronger inhibition in neonate animals than the adults. Together, we conclude that gluconate inhibits neonate seizure through inhibiting CLC-3 Cl^−^ channels, not Ca^2+^ currents.

After providing solid evidence on gluconate inhibition of neonatal seizures, we would like to caution on any attempt to jump on clinic applications before conducting more studies. For example, the working concentration of gluconate is quite high, with an IC_50_ of NaGluc at 8.6 mM in inhibiting CLC-3 Cl^−^ channels in neonatal hippocampal neurons in rodents. On the other hand, gluconate is widely used as an additive agent in food and medicine industry. For example, 10% calcium gluconate was used to treat hyperkalemia and hypocalcemia [53, 54], which is a very high concentration. Another factor to concern is how much gluconate can pass through the blood brain barrier (BBB). While we do not have direct data on this, it is worth to point out that the BBB in neonates may not be as restrictive as that in the adults [55]. Furthermore, some studies also report that BBB may be leakier in the epileptic brains [56, 57]. In fact, as a monocarboxylate, gluconate may cross neonatal BBB through the monocarboxylate transporter [58], but more studies are needed to test the efficacy directly.

In conclusion, our studies have identified a novel anti-epileptic agent gluconate that can inhibit neonatal seizures by blocking CLC-3 Cl^−^ channels. Because gluconate is already used as a food and drug additive for human consumption, our findings may lead to a novel therapy to treat neonatal seizures. On the other hand, since this is only the first study revealing gluconate effect on neonatal seizures in rodents, there are many more studies required to investigate the pre-clinical effects of gluconate on large animals such as non-human primates. Furthermore, before starting any phase I human clinical trial, toxicology studies together with pharmacodynamics and pharmacokinetics studies are necessary to test the safety and dosage of gluconate first in animals. Therefore, this study is a starting point rather than the endpoint toward a potential new clinical therapy.

## Materials and methods

### Animals

C57BL/6 J mice were purchased from the Jackson Laboratory. The *Clcn3*^*−/−*^ mice were generated by replacing part of exon 6 and whole exon 7 with a cassette containing the neomycin resistance gene [18, 20, 28]. The majority of experiments were performed at Penn State University. The animal protocol was approved by the Pennsylvania State University IACUC in accordance with the National Institutes of Health Guide for the Care and use of Laboratory Animals. For in vivo experiments on adult mice or neonatal rats, the procedures were approved by the Committee of Animal Use for Research and Education of Fudan University and South China Normal University, respectively, in accordance with the ethical guidelines for animal research. Animal rooms were automatically controlled at 12 h light/dark cycle, and water and food were available ad libitum.

### Cell culture and transfection

Mouse cortical neurons were prepared from newborn C57BL/6 J mice as previously described [25]. Briefly, the newborn mouse cerebral cortices were dissected out in ice-cold HEPES-buffered saline solution, washed and digested with 0.05% trypsin-EDTA at 37 °C for 20 min. After deactivation of trypsin with serum-containing medium, cells were centrifuged, resuspended, and seeded on a monolayer of cortical astrocytes at a density of 10,000 cells/cm2 in 24-well plates. The neuronal culture medium contained MEM (500 ml, Invitrogen), 5% fetal bovine serum (Atlanta Biologicals), 10 ml B-27 supplement (Invitrogen), 100 mg NaHCO_3_, 2 mM Glutamax (Invitrogen), and 25 units/ml penicillin and streptomycin. AraC (4 μM, Sigma) was added to inhibit the excessive proliferation of astrocytes. Cell cultures were maintained in a 5% CO_2_-humidified incubator at 37 °C for 14–21 days.

Human embryonic kidney (HEK) 293 T cells were maintained in DMEM supplemented with 10% FBS and 25 units/ml penicillin/streptomycin. PEI kit (molecular weight 25,000, Polysciences, Inc.) was applied for HEK cell transfection. In brief, 1 μg DNA was diluted into 50 μl of OptiMEM (Invitrogen), then mixed with 4 μl of PEI (1 μg/ μl), incubated for 5 min, and added drop-by-drop to the culture well containing 500 μl of medium. After 5 h incubation, the transfection reagents were washed off by fresh culture medium. Two days after transfection, HEK293T cells were used for electrophysiological study. Rat CLC-3 short transcript fused to eGFP plasmid (pCLC3sGFP) was purchased from Addgene (plasmid # 52423, Steven Weinman) [29].

### Cell viability assay

A LIVE/DEAD® Viability/Cytotoxicity Assay Kit (L3224, Life Technologies) containing ethidium homodimer-1 and calcein-AM was used to examine cell viability. Ethidium homodimer-1 binds to cellular DNA and typically labels dead cells in red fluorescence, while Calcein-AM can be cleaved by esterases in live cells to give strong green fluorescence. After drug treatment, neurons were incubated in bath solution containing 1 μM calcein-AM and 4 μM ethidium homodimer-1 at room temperature for 40 min. Cell survival and death rate were measured by quantifying the percentage of green and red fluorescent cells, respectively. For each group, at least 5 fields of each coverslip were imaged for data analysis.

### Mouse brain slice preparation

Brain slices were prepared from C57BL/6 J mice (male and female). Animals were anesthetized with Avertin (tribromoethanol, 250 mg/kg) and decapitated. Hippocampal horizontal sections (300–400 μm) were prepared by Leica VT1200S vibratome. The neonatal (P6–12) and young adult (P21–33) brain slices were cut in ice-cold artificial cerebral spinal fluid (aCSF) (in mM): 125 NaCl, 26 NaHCO_3_, 10 glucose, 2.5 KCl, 2.5 CaCl_2_, 1.25 NaH_2_PO_4_, and 1.3 MgSO_4_, osmolarity 290–300 mOsm, aerated with 95% O_2_/5% CO_2_. Slices were then transferred to incubation chamber containing normal aCSF saturated with carbogen (95% O_2_/5% CO_2_) at 33 °C for 30 min, followed by recovery at room temperature for 1 h before use. The adult brain slices (> 2 months old) were prepared following previously described methods [59], with some additional modifications based on NMDG (N-Methyl-D-glucamine) recovery method. After anesthetized with Avertin, the adult mice was perfused with NMDG cutting solution (in mM): 93 NMDG, 93 HCl, 2.5 KCl, 1.25 NaH_2_PO_4_, 30 NaHCO_3_, 20 HEPES, 12 N-Acetyl-L-cysteine, 15 Glucose, 5 Sodium ascorbate, 2 Thiourea, 3 Sodium pyruvate, 7 MgSO_4_, 0.5 CaCl_2_, then the brain was removed and cut in the NMDG cutting solution at room temperature. The brain slices were kept at 32–34 °C in oxygenated NMDG solution for 10–15 min. Slices were then transferred to the modified HEPES holding aCSF (in mM): 92 NaCl, 2.5 KCl, 1.25 NaH_2_PO_4_, 30 NaHCO_3_, 20 HEPES, 12 N-Acetyl-L-cysteine, 15 Glucose, 5 Sodium ascorbate, 2 Thiourea, 3 Sodium pyruvate, 2 MgSO_4_, 2 CaCl_2_, for about 0.5–1 h at room temperature before recording. Individual slices were transferred to a submerged recording chamber where they were continuously perfused (2–3 ml/min) with normal aCSF saturated by 95% O_2_/5% CO_2_ at 31–33 °C (TC-324B, Warner instruments Inc). Slices were visualized with infrared optics using an Olympus microscope equipped with DIC optics.

### Electrophysiology

#### Whole-cell recording in cell cultures

The cultured neurons were placed in the recording chamber with continuous perfusion of the bath solution consisting of (mM): 128 NaCl, 10 Glucose, 25 HEPES, 5 KCl, 2 CaCl_2_, 1 MgSO_4_, pH 7.3 adjusted with NaOH, and osmolarity ~ 300 mOsm. For recording spontaneous firing under current clamp mode, pipettes were filled with an internal solution containing (in mM): 125 K-gluconate, 5 Na-phosphocreatine, 5 EGTA, 10 KCl, 10 HEPES, 4 Mg-ATP, 0.3 Na-GTP, 280–290 mOsm, pH 7.3 adjusted with KOH. Epileptiform activity in cultured neurons was induced either by 10 μM cyclothiazide (CTZ) for 24 h, or 1 μM KA for 2 h, or 50 μM 4-AP for 2 h. The burst activity was defined as previously described [25]. In brief, bursts must contain at least five consecutive action potentials overlaying on top of the large depolarization shift (≥10 mV depolarization and ≥ 300 ms in duration).

#### Brain slice recording

Field potential and cell-attached recordings were performed with glass electrodes (2–4 ΜΩ tip resistance) filled with external solution. The field potential recording electrode was placed into the CA3 pyramidal layer. For current clamp (I = 0) recordings, the amplifier was set at the 100x with a band pass filter of 0.1–5 KHz. All recordings were performed at 31–33 °C. The epileptic activity was evoked by Mg^2+^-free aCSF, or addition of 50 μM 4-AP or 8.5 mM K^+^ in the aCSF. The paroxysmal discharges were defined as in the previous study [33].

For Cl^−^ current recording, 0 Ca^2+^ pipette solution contained either high or normal Cl^−^ were used. High Cl^−^ pipette solution (mM): 135 CsCl, 10 HEPES, 5 EGTA, 5 TEACl, 4 MgATP (pH 7.3 adjusted with CsOH, 280–290 mOsm); Normal Cl^−^ pipette solution (mM): 125 Cs-Acetate, 10 CsCl, 10 HEPES, 5 EGTA, 5 TEA-Cl, 4 MgATP (pH 7.3 adjusted with CsOH, 280–290 mOsm). To isolate Cl^−^ current: (1) Extracellular Na^+^ was replaced by NMDG^+^ and voltage-gated Na^+^ channels were blocked by TTX; (2) K^+^ and Ca^2+^ were removed from bath solution; (3) K^+^ channels were blocked with Cs^+^ and tetraethylamonium (TEA) in the pipette solution, and 4-AP in the bath solution; (4) CdCl_2_ was added into bath solution to block Ca^2+^ channels; (5) Picrotoxin (50 μM) was included to block GABA_A_ receptors. Thus, the external solution contained the following (in mM): 135 NMDG-Cl, 20 HEPES, 20 Glucose, 5 4-AP and 2 MgSO_4_, supplemented with 1 μM TTX, 200 μM CdCl_2_, and 50 μM picrotoxin, osmolarity ~ 300 mOsm, pH 7.3–7.4 after aerated with 95% O_2_/5% CO_2_. Voltage steps from − 80 to + 90 mV (10 mV increments) were applied from holding potential of 0 mV for high Cl^−^ pipette solution [60, 61] and voltage steps from − 100 to + 30 mV (10 mV increments) were applied from holding potential of − 60 mV for normal Cl^−^ pipette solution. Data were collected with a MultiClamp 700A amplifier and pCLAMP 10 software (Molecular Devices).

For gramicidin-perforated whole-cell recordings, the KCl pipette solution was used (mM): 135 KCl, 10 HEPES, 2 EGTA, 4 MgATP, and 0.3 Na-GTP (pH 7.3), osmolarity 290–300 mOsm. Pipette tip was first filled with gramicidin-free KCl internal solution and then back filled with this internal solution containing gramicidin (40 μg/ml). To measure GABA_A_ receptor (GABA_A_R) reversal potential (*E*_GABA_), focal pressure ejection of GABA_A_R agonist isoguvacine (100 μM) via a glass pipette controlled by a Picrospitzer (50 ms puff at 10 psi) was used to activate GABA_A_-Rs on CA3 pyramidal neurons with gramicidin-perforated patch (Ra ≤ 80 MΩ) under voltage-clamp at different holding potentials. TTX (1 μM), CNQX (10 μM) and AP5 (50 μM) were added both in the bath and puffer solution to avoid any glutamate currents. The holding potential and peak amplitude were plotted and the *E*_GABA_ was determined for each cell.

#### Power analysis

Hamming window function was applied before power spectrum analysis. Power was calculated by integrating the root mean square value of the signal in frequency bands from 0.1 to 1000 Hz in sequential 5-min time windows before, during, and after drug applications. To avoid slice-to-slice and electrode contact variability, power values were normalized to control condition before drug application for each slice, and then averaged across different slices for statistical analysis.

### Immunostaining and Western blot

#### Immunostaining

The neonatal (P12, male and female) and adult (3–4 months, male) mice were anesthetized by Avertin (tribromoethanol, 250 mg/kg, i.p.) and sequentially perfused with ice-cold aCSF and 4% paraformaldehyde (PFA) in PBS. Brains were collected and postfixed with 4% PFA overnight at 4 °C. Coronal sections of 40 μm thickness were cut by a vibratome (VT1000, Leica, Germany) for immunohistochemistry. For some experiments, brain slices were prepared following the electrophysiology protocol described above, with 200 μm thickness for immunostaining and 400 μm thickness for Western blot. In general, slices were recovered for 1 h at room temperature and then randomly divided into two groups. One group of slices were incubated in normal aCSF for 1 h at 33 °C. Another group of slices were incubated in 0 Mg^2+^ aCSF for 1 h at 33 °C to induce epileptiform activity. Slices were then fixed by 4% PFA overnight at 4 °C. For CLC-3 staining, slices were pretreated with blocking solution (0.3% Triton-X and 5% normal donkey and goat serum in 0.1 M PBS) for 2 h, and then incubated for 48–72 h with CLC-3 primary antibody (Rabbit, 1:500, Alomone Labs, ACL-001). Some slices were incubated with rabbit IgG as control. After washing three times in PBS with 0.01% triton-X, the brain sections were incubated with goat anti rabbit secondary antibodies conjugated to Cy3 (1:500, Jackson ImmunoResearch) for 2 h at room temperature. The brain sections were mounted on a glass slide with an anti-fading mounting solution (Invitrogen). Fluorescent images were acquired on a confocal microscope system (Olympus FV1000, Japan or Zeiss LSM 880 with airyscan, German). For each slice, at least 2–3 fields of CA3 region were imaged. To quantify CLC-3 fluorescent intensity in CA3 pyramidal layer, confocal images were analyzed using NIH Image J software.

#### Western blot

Cell surface biotinylation of mouse brain slices was conducted as published [62]. After euthanization, mouse brains were freshly isolated and sliced into 1 mm coronal sections. Brain slices were incubated in 1 mg/ml sulfo-NHS-SS-biotin (ThermoFisher) in pre-chilled oxygenated aCSF on ice for 30 min and then washed twice with 50 mM glycine and three times with 1 mg/ml BSA in pre-chilled oxygenated aCSF. Hippocampus was dissected from slices, extracted and the biotinylated proteins purified using NeutrAvidin beads (ThermoFisher) and processed for Western blot with following antibodies: rabbit anti CLC-3 (Rabbit, 1:1000, Alomone, ACL-001), Rabbit anti-Na-K ATPase (1:10,000, Abcam, ab76020). Blots were developed with goat anti-mouse IRDye 680RD and goat anti-rabbit IRDye 800CW (1:5000, LI-COR) and Odyssey CLx imager (LI-COR) and quantitated using Image Studio (LI-COR).

### Electroencephalogram (EEG) recording and analysis

To test the in vivo anti-epileptic effects of gluconate in neonatal and adult rodents, P8–12 Sprague-Dawley rats or 2-month old male C57BL/6 mice were deeply anesthetized with pentobarbital sodium (50 mg/kg for neonatal rat and 100 mg/kg for adult mice, intraperitoneal injection). Two stainless steel screws (1 mm in diameter) were inserted into the skull above the cortex as EEG recording electrodes, one ground electrode as well as one reference electrode were located + 1.8 mm anterior to bregma, ± 0.5 mm lateral to the midline, and 1 mm below the cortical surface. All electrodes were attached to a micro-connector and fixed onto the skull with dental cement. After surgery, neonatal rats were returned to their mothers and allowed to recover for 2 days prior to subsequent EEG recording. The adult mice were single housed in order to prevent damage of the implanted electrodes and allowed to recover at least 5 days before EEG recording. The baseline of EEG was recorded for 0.5–1 h to allow the animal to adapt to the environment.

To induce seizure activity, kainic acid (KA, 2 mg/kg) was administered intraperitoneally (i.p.) into the neonatal rat. D-gluconic acid sodium salt (2 g/kg) or 0.9% saline was i.p. injected at 10 min after KA administration. In adult mice, KA (10 mg/kg) was i.p. administered to induce seizure burst activity, and D-gluconic acid sodium salt (2 g/kg) or 0.9% saline was i.p. injected at 10 min after KA administration. Epileptiform activity was monitored for 2 h after KA injection. After the experiment, animals were injected with diazepam to protect the animals from recurrent seizures. The experiments on hypoxia-ischemia induced seizure model included both male and female neonatal rats (Albertsson and Wang, 2015; Zayachkivsky et al., 2015). Rat pups were anesthetized by isofluorane (5.0% for induction and 1.0% for maintenance). The right common carotid artery of neonatal rats was ligated permanently at P5 for ischemia induction. After 3 days recovery, the EEG recording electrodes were implanted in the rat skull at P8. The EEG recording and hypoxia stimulation was performed at P10 (Additional file 1: Figure S6). The electrophysiological signals were amplified (1000x) and filtered ](0–500 Hz) with a NeuroLog System (Digitimer Ltd., Hearts, UK) and visualized and stored in a PC through a D-A converter, CED 1401 micro (Cambridge Electronic Design, Cambridge, UK). The power level of different frequency components in the neonatal EEG signal was examined by power spectrum analysis. Power was calculated in 5-min time windows by root mean square amplitude from 1 to 100 Hz (EEG band). Seizures were defined as electrographic seizure activity recorded from right hemisphere, only when they were consisted of paroxysmal rhythmic spikes of high amplitude, diffuse frequency of > 8 Hz, lasting > 3 s [63].

### Data analysis

Data were shown as mean ± s.e.m. Student’s *t*-test (paired or unpaired) and Mann-Whitney test were performed for two-group comparison, and the χ^2^ test was used to compare the difference of percentage between two groups. For comparison among multiple groups, one-way ANOVA followed with post hoc tests were used. Statistical significance was set at *P* < 0.05.

## Additional file

Additional file 1: **Figure S1.** Gluconate showed no effect on cation channels in neuronal cultures. **Figure S2.** Gluconate inhibits the Cl^−^ currents recorded with physiologic [Cl^−^] in the pipette solution. **Figure S3**. Gluconate inhibits CLC-3 channel-mediated Cl^−^ currents in HEK293T cell. **Figure S4.** Broad inhibition of NaGluc on epileptiform activity induced by various epileptic stimuli in neonatal hippocampal slices. **Figure S5.** β-HB inhibits CLC-3 channels and epileptiform activity in neonatal slices. **Figure S6.** Illustration of the procedure of hypoxia-ischemia induced neonatal epilepsy model. **Figure S7.** Synergistic effect between gluconate and phenobarbital on hypoxia-ischemia induced neonatal seizure activity in vivo*. (DOCX 6978 kb)*

## Abbreviations

AEDs: Antiepileptic drugs
Bum: Bumetanide
CTZ: Cyclothiazide
DIDS: 4,4′-Diisothiocyanato-2,2′-stilbenedisulfonic acid disodium salt
EEG: Electroencephalogram
*E*_GABA_: Reversal potential of GABA currents
EthD-1: ethidium homodimer-1
GABA: Gamma-Aminobutyric Acid
HI: Hypoxia-ischemia
IC_50_: The half maximal inhibitory concentration
KA: Kainic acid
KCC2: K^+^-Cl^−^ cotransporter 2
NaGluc: Sodium Gluconate
NKCC1: Na^+^-K^+^-Cl^−^ cotransporter 1
NPPB: 5-Nitro-2-(3-phenylpropylamino) benzoic acid
PCR: Polymerase chain reaction
β-HB: β-Hydroxybutyric acid
