# Supplementary material for: Gluconate suppresses seizure activity in developing brains by inhibiting CLC-3 chloride channels
Source: Mol Brain. 2019 May 15;12:50. doi: 10.1186/s13041-019-0465-0 (PMC6518791; doi:10.1186/s13041-019-0465-0)
Supplement: Supplementary file 1 — Figure S1. Gluconate showed no effect on cation channels in neuronal cultures. Figure S2. Gluconate inhibits the Cl− currents recorded with physiologic [Cl−] in the pipette solution. Figure S3. Gluconate inhibits CLC-3 channel-mediated Cl− currents in HEK293T cell. Figure S4. Broad inhibition of NaGluc on epileptiform activity induced by various epileptic stimuli in neonatal hippocampal slices. Figure S5. β-HB inhibits CLC-3 channels and epileptiform activity in neonatal slices. Figure S6. Illustration of the procedure of hypoxia-ischemia induced neonatal epilepsy model. Figure S7. Synergistic effect between gluconate and phenobarbital on hypoxia-ischemia induced neonatal seizure activity in vivo. (DOCX 6978 kb) [file 13041_2019_465_MOESM1_ESM.docx]

**Supplementary Info for:**

**Gluconate suppresses seizure activity in developing brains**

**by inhibiting CLC-3 chloride channels**

**Authors:** Zheng Wu^1,^ ^†^, Qingwei Huo^2,6, †^, Liang Ren^3^, Fengping Dong^1^, Mengyang Feng, Yue Wang^1^, Yuting Bai^1^, Bernhard Lüscher, Sheng-Tian Li^4^, Guan-Lei Wang^5^, Cheng Long^2^, Yun Wang^3^, Gangyi Wu^1,2^, Gong Chen^1, *^

*Editorial correspondence should be addressed to:

Gong Chen, Ph.D.

Professor and Verne M. Willaman Chair in Life Sciences

Department of Biology, Huck Institutes of Life Sciences,

The Pennsylvania State University, University Park, PA 16802, USA.

Email: [gongchen@psu.edu](mailto:gongchen@psu.edu)**;** Phone: 814-865-2488

Website: <http://bio.psu.edu/directory/guc2>

†Contribute equally to this study.

Supplementary Figures and Legends:


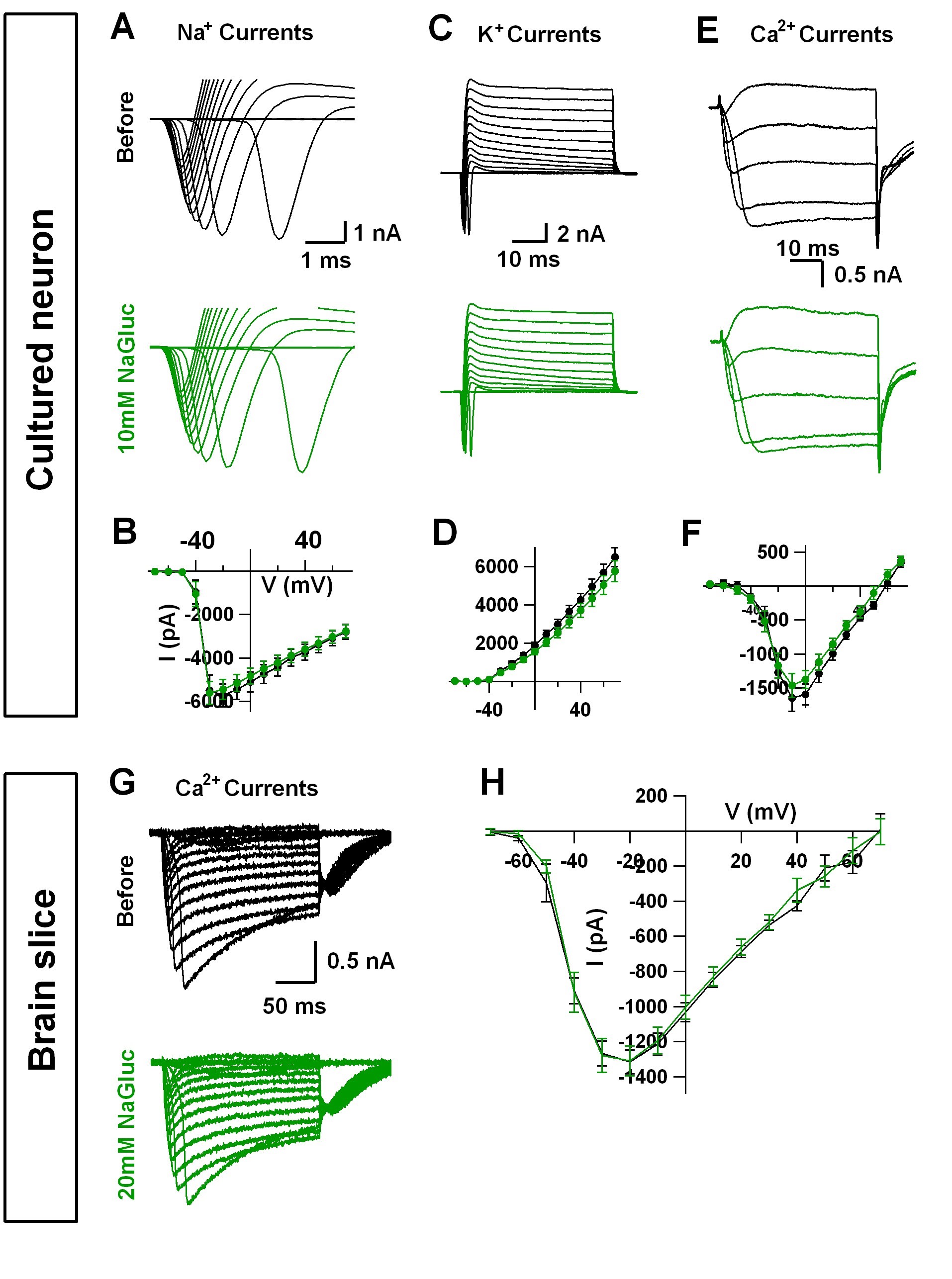


**Figure S1. Gluconate showed no effect on cation channels in neuronal cultures.** (**A**) Representative Na^+^ current traces before and during the application of NaGluc (10 mM) in the cultures. (**B**) NaGluc (green) showed no effect on the I-V curve of whole-cell Na^+^ currents in cultured mouse cortical neurons. (**C, D**) NaGluc (green) showed no effect on the K^+^ currents in the cultures. (**E, F**) NaGluc (green) showed no obvious effect on the Ca^2+^ currents in the cultures. (**G, H**) NaGluc (green) showed no effect on the Ca^2+^ currents in the CA3 pyramidal neurons in hippocampal slices. Data are mean ± s.e.m.


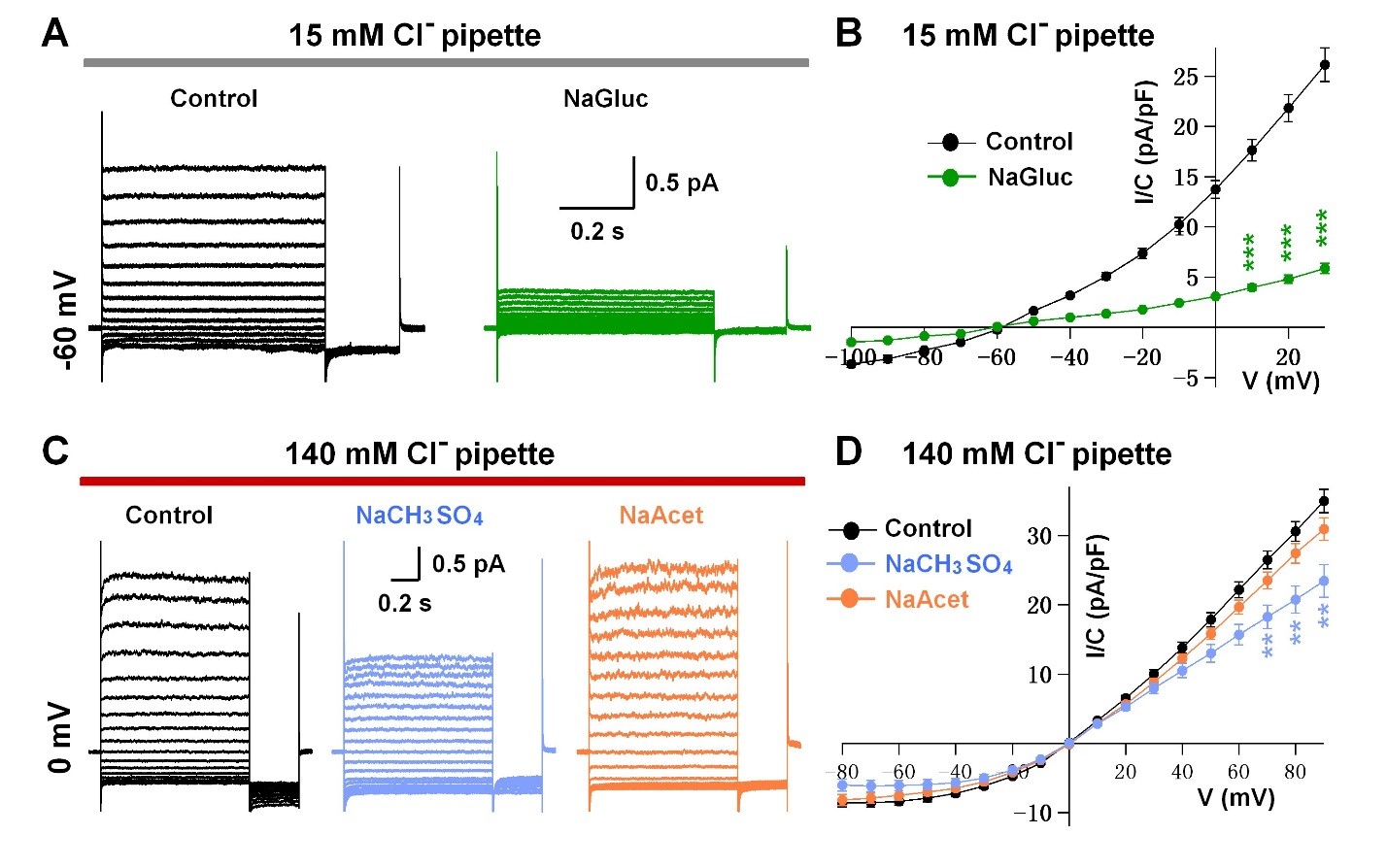


**Figure S2. Gluconate inhibits the Cl^-^ currents recorded with physiologic [Cl^-^] in the pipette solution.**

(**A**) Representative Cl^-^ currents recorded with near physiological concentration of Cl^-^ in the pipette solution under control (left) or NaGluc treatment (20 mM, right). The acetate was used to replace the rest of the Cl^-^ in the pipette solution. (**B**) I-V plot showing the outward rectifying Cl^-^ currents with low Cl^-^ in the pipette solution and a significant inhibitory effect by NaGluc. (**C**) Typical voltage-gated Cl^-^ currents recorded with high concentration of Cl^-^ in the pipette solution. Bath application of sodium methanesulfonate (NaCH_3_SO_3_, 20 mM) inhibited Cl^-^ currents but sodium acetate (NaAcet, 20 mM) showed no effect. This is why we chose acetate to replace Cl^-^ for the low Cl^-^ pipette solution. (**D**) I-V plot of Cl^-^ currents showing the effects of 20 mM NaAcet (orange) or 20 mM NaCH3SO3 (blue). Data are mean ± s.e.m., ** *P* < 0.01, *** *P* < 0.001.

**
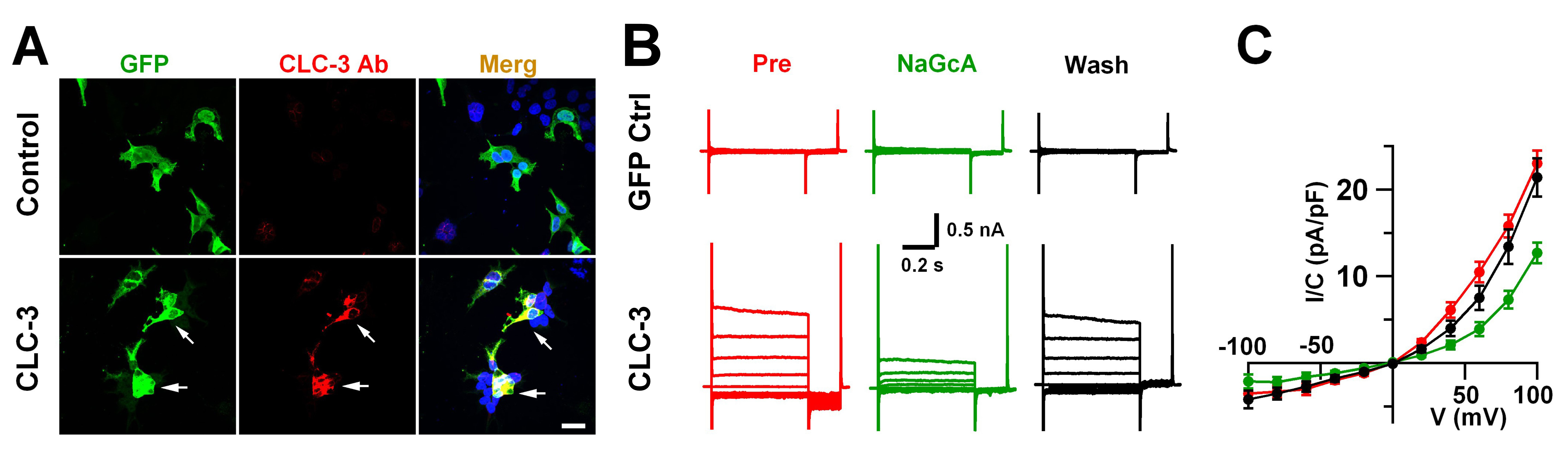
**

**Figure S3. Gluconate inhibits CLC-3 channel-mediated Cl^-^ currents in HEK293T cell.**

(**A**) Expression of CLC-3 Cl^-^ channels in HEK293T cells. Top row, overexpressing EGFP alone as a control. Bottom row, overexpressing CLC-3-EGFP Cl^-^ channels in HEK293T cells. CLC-3 Cl^-^ channels were detected by immunostaining with CLC-3 antibodies (red). Scale bar, 40 μm. (**B**) Top row, EGFP transfected cells did not show Cl- currents. Bottom row, large Cl^-^ currents were recorded from CLC-3-EGFP transfected HEK293T cells. Application of 20 mM NaGluc significantly inhibited the CLC-3 channel-mediated Cl^-^ currents (green traces, middle). (**C**) I-V curves showing the NaGluc inhibition of CLC-3 channel-mediated Cl^-^ currents (CLC-3, 23.0 ± 1.5 pA/pF, *n* = 7; CLC-3 + NaGluc, 12.7 ± 1.2 pA/pF, *n* = 7; *P* < 0.004, paired *t*-test; HP = +90 mV). Data are shown as mean ± s.e.m.


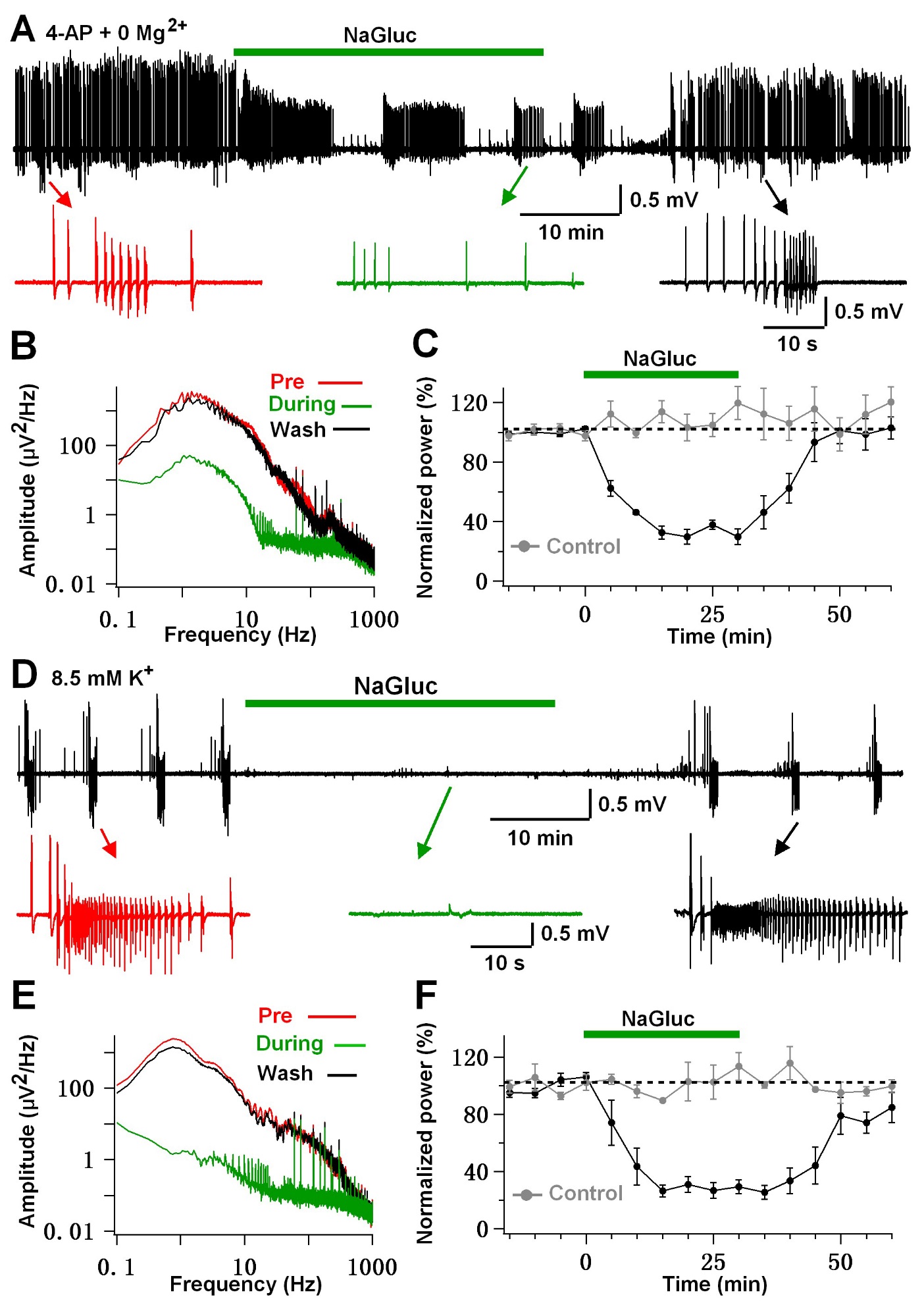


**Figure S4. Broad inhibition of NaGluc on epileptiform activity induced by various epileptic stimuli in neonatal hippocampal slices.** (**A**) Typical field potential recording showing epileptiform activity induced by 50 μM 4-AP in the CA3 pyramidal layer of neonatal hippocampal slices, and its inhibition by NaGluc (20 mM). (**B**) Power spectra of epileptiform activity before (red), during (green), and after (black) NaGluc application. Note that NaGluc (green) significantly reduced the power amplitude. (**C**) Normalized power (in 5-minute time windows) showing the time course of the inhibition of NaGluc on the epileptiform activity induced by 4-AP. (**D**) NaGluc (20 mM) greatly suppressed the epileptiform activity induced by high K^+^ in neonatal hippocampal slices. (**E**) Power spectra showing a significant reduction of epileptiform activity during NaGluc application. (**F**) Normalized power illustrating the time course of NaGluc inhibition on the epileptiform activity induced by high K^+^ aCSF. The grey line in panels **C** and **F** are controls, representing the 20 mM NaCl effect on neonatal (P8-12) epileptiform activity. Data are shown as mean ± s.e.m.


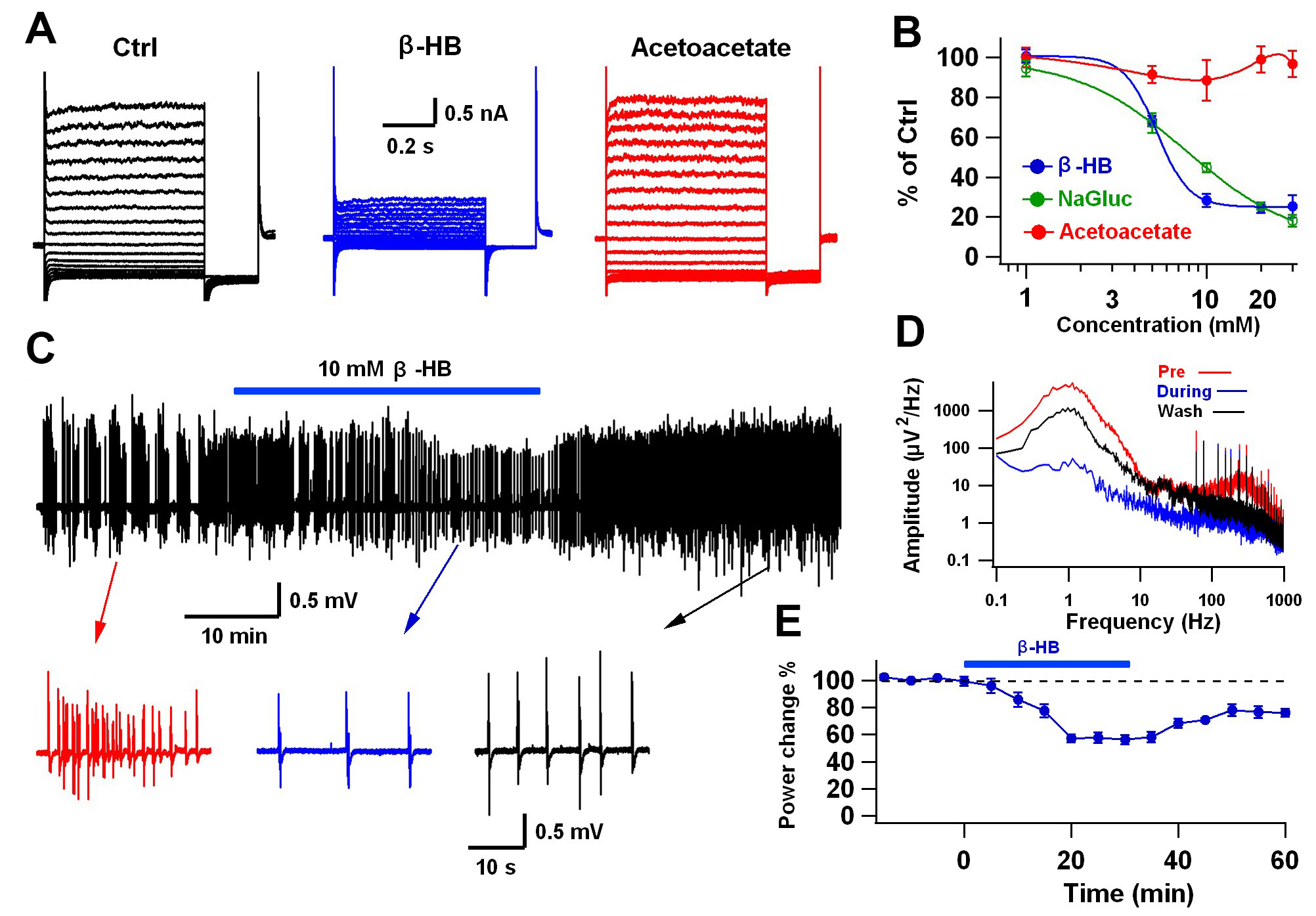


**Figure S5. β-HB inhibits CLC-3 channels and epileptiform activity in neonatal slices.** (**A**) Representative Cl^-^ current recorded under control (black), 10 mM β-HB (blue, H0231, TCI) and 10 mM acetoacetate (red, A1478, TCI). (**B**) Normalized Cl^-^ current dose-response curve of β-HB (blue), acetoacetate (red) and gluconate (green). (**C**) Typical epileptiform activity induced by 0 Mg^2+^ aCSF with the energy source switched from 10 mM glucose to 10 mM β-HB. The enlarged insets (bottom) showed the frequency of epileptiform discharge decreased in presence of β-HB (blue). (**D**) Power spectra of epileptiform activity (5-minute time windows) before (black), during (blue), and after (red) β-HB application. (**E**) Normalized power showing the time course of β-HB (10 mM) inhibition on the epileptiform activity induced by 0 Mg^2+^ aCSF. Data are shown as mean ± s.e.m.


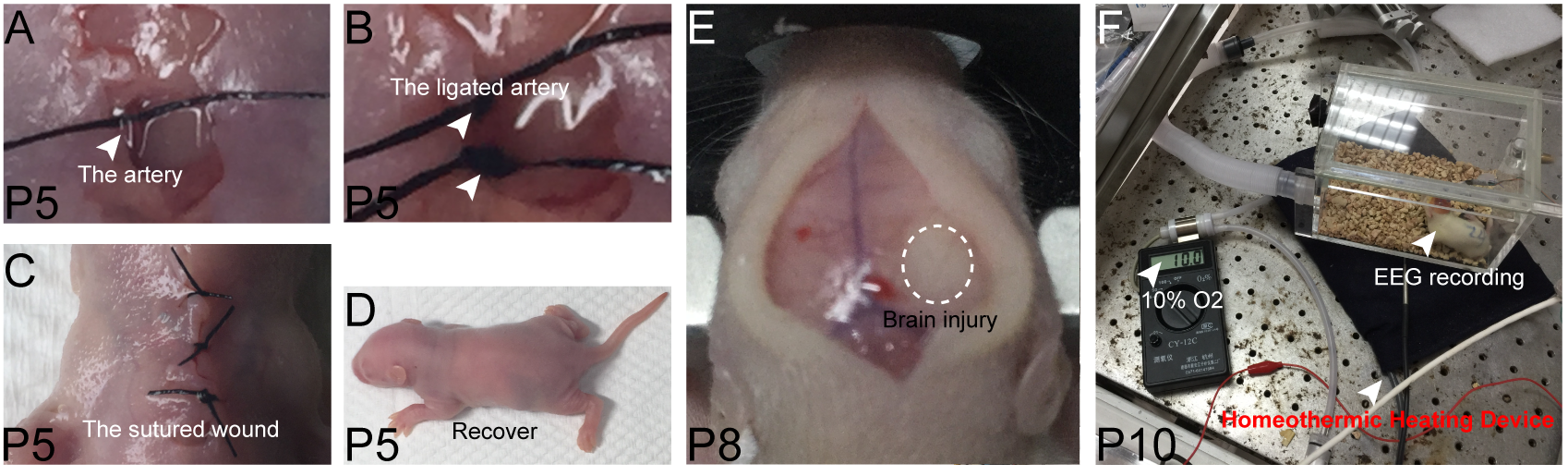


**Figure S6. Illustration of the procedure of hypoxia-ischemia induced neonatal epilepsy model.** (**A-D**) The right common carotid artery was ligated at P5 to induce ischemia. (**E**) After 3 days of recovery, the secondary surgery was performed for the implantation of EEG recording electrodes. The ischemia-induced injury area (dashed line circle) was clearly observed at P8 when implanting the electrodes. (**F**) At P10, EEG recording was performed and 10% O_2_ was given to the neonatal rats to induce epileptiform activity.

Related to Figure S7.

**
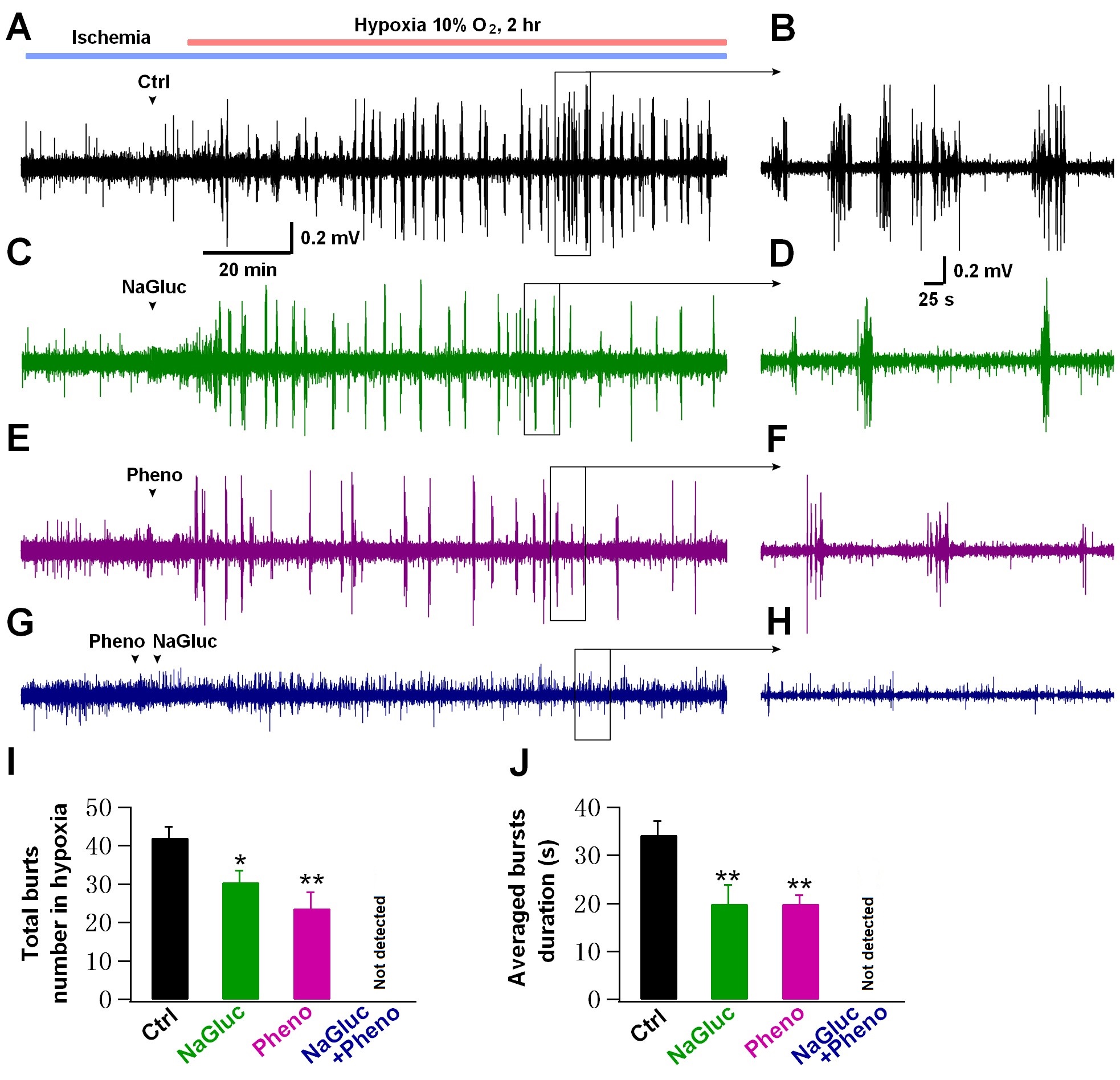
**

**Figure S7. Synergistic effect between gluconate and phenobarbital on hypoxia-ischemia induced neonatal seizure activity *in* *vivo.*** (**A**) Representative EEG recording showing recurrent seizure burst activities from a P10 rat after ischemia-hypoxia stimulation. Arrowhead indicates saline injection as a control (0.1 ml/10 g, i.p.; 5 min before 10% O_2_ application). (**B**) Expanded view of seizure burst discharges from the box in **A**. (**C, D**) Representative EEG trace (P10 rat) showing the seizure activity significantly reduced by NaGluc administration (2 g/kg, i.p.; injected 5 min before hypoxia). (**E, F**) Typical EEG trace showing the effect of phenobarbital (25 mg/kg, i.p.) on the seizure bursts induced by ischemia-hypoxia in a neonatal rat (P10). (**G, H**) Combined administration of NaGluc (2 g/kg) and phenobarbital (25 mg/kg) showed synergistic inhibition of the seizure activity in neonatal rats (P10). (**I, J**) Summarized data showing the effect of NaGluc, phenobarbital, or their combination on the total number of bursts (**I**) or burst duration (**J**) in neonatal rats (ctrl animals, n = 10; NaGluc, n = 8; phenobarbital, n = 7) (one-way ANOVA followed with Tukey post hoc test). Note that no seizure bursts were detected in NaGluc + phenobarbital group (n = 8). Data are shown as mean ± s.e.m., *P < 0.05, ** P < 0.01.
